# Persistent association between family socioeconomic status and primary school performance in Britain over 95 years

**DOI:** 10.1038/s41539-022-00120-3

**Published:** 2022-04-20

**Authors:** Sophie von Stumm, Sophie Nicole Cave, Paul Wakeling

**Affiliations:** Department of Education, University of York, YO10 5DD Heslington, York UK

**Keywords:** Education, Human behaviour

## Abstract

In Britain and elsewhere, the influence of family socioeconomic status (SES) on education is already evident in primary school, and it persists and increases throughout the school years, with children from impoverished families earning lower grades and obtaining fewer educational qualifications than children from more privileged backgrounds. Reducing the effect of family background on children’s education is a pivotal aim of educators, policymakers, and researchers, but the success of their efforts is poorly evidenced to date. Here, we show for the first time that over 95 years in Britain the association between family SES and children’s primary school performance has remained stable. Across 16 British population cohorts born between 1921 and 2011 (*N* = 91,935), we confirmed previous findings of a correlation between family SES and children’s school performance of 0.28 [95% Confidence Interval 0.22–0.34], after adjusting for cohort-specific confounders. Contrary to the popular assumption that family background inequality has increased over time, we observed only minimal differences in the association between family SES and school performance across British cohorts. We argue that education policies must prioritize equity in learning outcomes over equality in learning opportunities, if they seek to disrupt the perpetuation of social and economic inequality across generations. We speculate that the influence of family SES on children’s education will only noticeably weaken if primary education settings become better equipped to meet and remediate the children’s differential learning needs.

## Introduction

A family’s socioeconomic status (SES) describes their level of access to and control over economic and social resources relative to that of other families. Family SES has been recognized as an important determinant of children’s educational opportunities and outcomes^1–4^, but less is known about the stability of the influence of family SES on children’s school performance over longer historical periods^5–8^.

Family SES captures a multitude of factors that interact to shape children’s neurocognitive development through synergistic biological pathways^9–11^, although it is typically operationalized by parents’ educational attainment, occupation, and income status^12,13^. A family’s SES represents their economic capital, for example, their access to good nutrition, high-quality housing, and safe transport; their cultural capital, including the competencies, skills, values, and aspirations that the family holds; and their social capital, which refers to a family’s network of personal and professional relationships^14,15^. These characteristics of family SES are embedded within a wider ecological framework that spans—among other things—the neighborhood, local labor markets, and the quality of nearby schools^16^. The influence of family SES on their offspring’s education emerges from children’s experiences within the family home and the conditions that inform their wider environment. For example, high SES parents have been found to talk on average more often with their children, using larger and more complex vocabularies, and referring to more abstract concepts than parents of lower SES^17–19^. In turn, children from high SES families develop greater verbal ability themselves^20^ and become more familiar with the language patterns and linguistic codes that prevail in formal educational settings and that are expected by teachers than low SES children^21,22^. The language environment that children experience is only one of many pathways through which family background exerts its pervasive influence on child development^9–11^. Yet, it exemplifies how children become differently equipped to meet the demands of formal education and to maximize its learning opportunities depending on their family’s SES.

The association between family background and school performance is evident before children start primary school^23,24^, and it persists over the course of compulsory education^25,26^. In fact, as children progress through school, the influence of their families’ background increases, and differences in school performance between high and low SES children magnify^2,26,27^. At the same time, children’s differences in school performance are highly stable from the first years of primary school through to the end of secondary school, with previous studies reporting correlations of 0.60 on average^26,28,29^. This finding implies that children who perform poorly at the beginning of formal schooling also tend to struggle throughout the primary and secondary school by comparison to the other students (i.e., rank-order stability). As a result, they are less likely to attain further educational qualifications after completing compulsory schooling and to experience the favorable life outcomes of children who perform well early on in school^27,30^. The rank-order stability of children’s differences in school performance across the school years is statistically independent of changes in the influence of their predictors. That is, how children perform in school relative to each other may not change across the duration of compulsory schooling, and yet the influence of family SES on children’s differences in school performance could be increasing, or decreasing, or remain stable. In societies that reward educational achievement, the combination of the rank-order stability and the widening of the SES-related differences in school performance begets the reproduction and perpetuation of social and economic inequality across generations^31^.

Educational policy in putatively meritocratic societies, like Britain, has traditionally focused on creating equal learning opportunities^32,33^. Equal educational opportunity cannot, however, translate into equal educational outcomes for children, who differ in their abilities to realize those opportunities^34,35^. To eliminate systematic inequality in educational outcomes, both learning opportunities and learning resources must be distributed so that students can achieve the same outcomes regardless of individual barriers and different starting conditions. An example of such (re)distribution efforts is awarding grants to schools, which enroll pupils from impoverished and unstable family homes to fund extra educational resources that these pupils need to overcome their disadvantages^36^. If this and similar policies were effective, a reduction in the influence of family background on children’s school performance should occur. Conversely, other education policies may, perhaps inadvertently, strengthen the relation between family SES and children’s education. For example, when school systems are highly differentiated, because they select students at relatively young ages into rigid academic and vocational tracks, the influence of family background increases, as does students’ inequality in educational outcomes^37,38^.

Besides the influences of educational policy, the association between family SES and children’s school performance is thought to have fortified because of two socio-political trends^5,7,8^. The first is the rise of socioeconomic inequality across the world that causes a growing gap in the access to and control over resources between the rich and poor^38^. The negative consequences of this development are particularly noticeable during periods of recession and austerity. Times of economic hardship, such as during the financial crisis of 2008 or the Covid-19 pandemic, are challenging for all members of society but their impact is most dramatic for low SES families with young children, who suffer the greatest risks for unemployment, financial debt, and social exclusion^39–42^. Children who grow up in low SES family homes during times of economic hardship are likely to experience significantly worse early life environments compared to low SES children who are born during more prosperous periods. As a result of the increased inequality in families’ socioeconomic resources, the association between family SES and children’s school performance may strengthen^5,7,43^.

A country’s economic climate may be somewhat independent of its contemporaneous educational policy. While public expenditure for education is unlikely to increase during times of economic hardship, governments may target their investments toward specific areas of the education sector, for example reducing achievement gaps in early years education versus widening participation in tertiary education. It is therefore possible that the effects of the economic climate and those of the respective education policies cancel out or reinforce each other in their influence on the relation between family SES and children’s education outcomes.

The second socio-political trend likely to strengthen the influence of family SES on children’s educational achievement is parents’ increasingly differential investment in their offspring’s learning. Students around the world engage now in a myriad of organized learning activities that take place outside formal classrooms (‘shadow education’), aimed at improving school performance^44^. The extent to which families can afford for their children to participate in these activities, for example, private tutoring, online courses, cram schools, and learning center franchises^45^, depends on their SES, as do their attitude towards, appreciation of, and involvement with them^22,44,46,47^. The effects of parents’ differential education investment on children’s school performance may be further magnified by policies that foster the market orientation of schools, for example, the introduction of league tables, parent representation on school governing bodies, and parent choice over which schools to send their children to^32^. Parents who are more invested in their children’s education will exhaust the opportunities that schools’ market orientation affords to elevate their offspring’s education, while children with less invested parents are unlikely to experience any benefits.

We report here an analysis of the stability of the association between family SES and children’s primary school performance in Britain over 95 years (preregistration https://osf.io/a8fwx/). We focused on primary school performance for three reasons. First, children’s differences in school performance are largely stable over time, with primary school performance predicting later school performance and long-term educational achievement^26–30^. Second, influences of family SES on education can be differentiated into ‘primary’ and ‘secondary’ effects, with the former referring to the association between SES and academic ability and the latter to other SES-related factors that inform educational choices and decision-making, like for example the affordability of tuition fees^48^. Primary school performance captures ‘primary effects’ in the reproduction of educational inequality, because primary schooling was state-funded and compulsory to attend across the periods and populations studied here, with negligible confounding due to ‘secondary’ effects. Third, primary school education has changed little in its structure and format over the past 150 years, by contrast to secondary and tertiary education across Britain. The principal goal of primary education—to equip children with the knowledge and skills essential for successfully participating in society (e.g., reading, writing, and arithmetic)—has remained the same since its inception^49^.

## Results

### Associations between family SES and children’s school performance

We identified 16 birth cohort studies that sampled representative populations from Britain and were born between 1921 and 2011 (see SI for detailed descriptions of all cohorts). Each population cohort recorded at least one marker of family SES, including parents’ education, occupation, or income, and one measure of children’s school performance during the primary school years (i.e., between the ages of 5 and 11 years), including teacher reported grades, school exam-based performance scores, and academic and cognitive ability test scores. After excluding those with missing data on family SES or school performance, sample sizes ranged from 240 to 14,923 across population cohorts, with a total of 91,935 individuals included in the analyses.

**Fig. 1:**
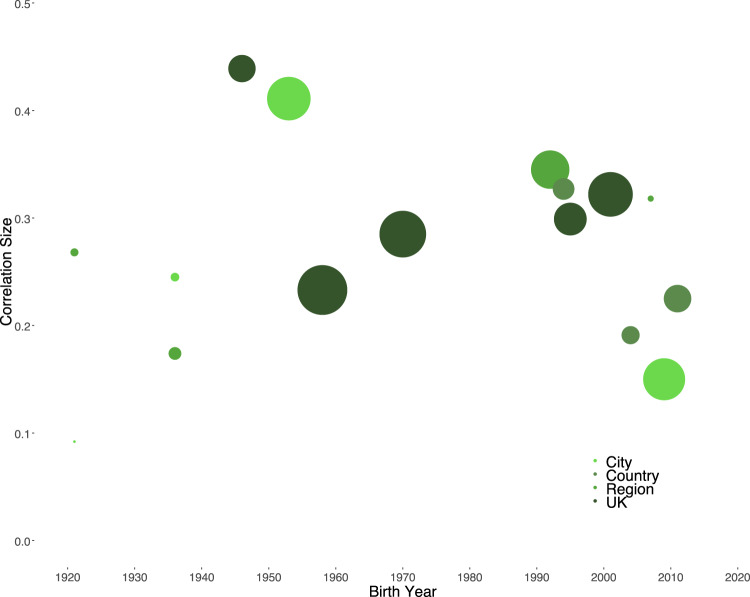
Raw correlations between family SES and children’s school performance across 16 cohorts born from 1921 through 2011. Note: The circles’ size represents the respective cohort’s sample size; the circles’ colors reflect the cohorts’ geographical scope. Pearson correlations were estimated.

We built standardized summary indices for family SES and school performance that were adjusted for the number of available markers per child in each cohort. Figure 1 shows the raw correlations between family SES and school performance plotted across the cohorts’ birth years from 1921 through 2011 (see also SI Table S2).

**Fig. 2:**
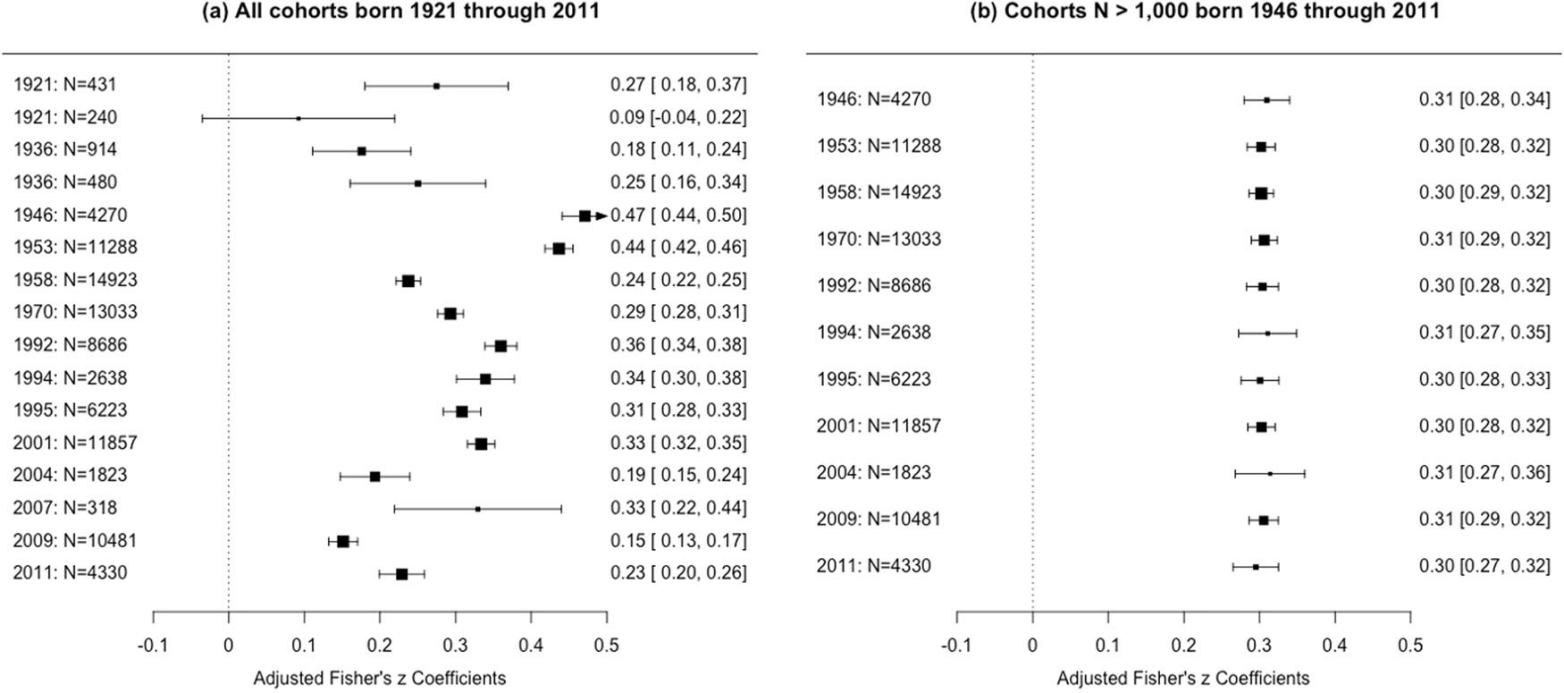
Forest plots of adjusted Fisher’s z-transformed correlations between family SES and children’s school performance over 95 and 70 years. Note. Cohorts are ordered by their average year of birth and shown with their respective sample sizes; Fisher’s z-transformed estimates are in the left column with [95% Confidence Interval]. Correlations were adjusted for cohort-specific confounders, including type of assessment of school performance; number of available indicators for SES and school performance; age of assessment of SES and school performance; cohorts’ geographical scope; and % of missing data. The size of the squares represents the cohort’s sample size. Data from the two cohorts born in 1921 and 1936, respectively, were not merged, because they differed in geographical scope.

With exception of the 1980s, representative population cohorts were available for all decades from the 1920s onwards (Fig. 1). After adjusting for cohort-specific confounders, correlations between family SES and school performance ranged from a minimum of 0.17 [95% Confidence Interval 0.10–0.23] to a maximum of 0.37 [0.28–0.46], as shown in panel (a) of Fig. 2. Across all 16 population cohorts, we estimated the average association between family SES and children’s school performance to be 0.28 [0.22–0.34], reflecting a medium effect size (see Table S2 in the SI for complete model results). By and large, the association between family SES and children’s school performance varied little and inconsistently across cohorts’ birth years. No systematic increasing or decreasing trends in the strength of the association could be observed across the past 95 years. Many of the larger differences in the associations occurred in cohorts born at the same time or in quick succession, suggesting that they are likely due to residual confounding from cohort-specific characteristics, rather than to meaningful time trends.

**Fig. 3:**
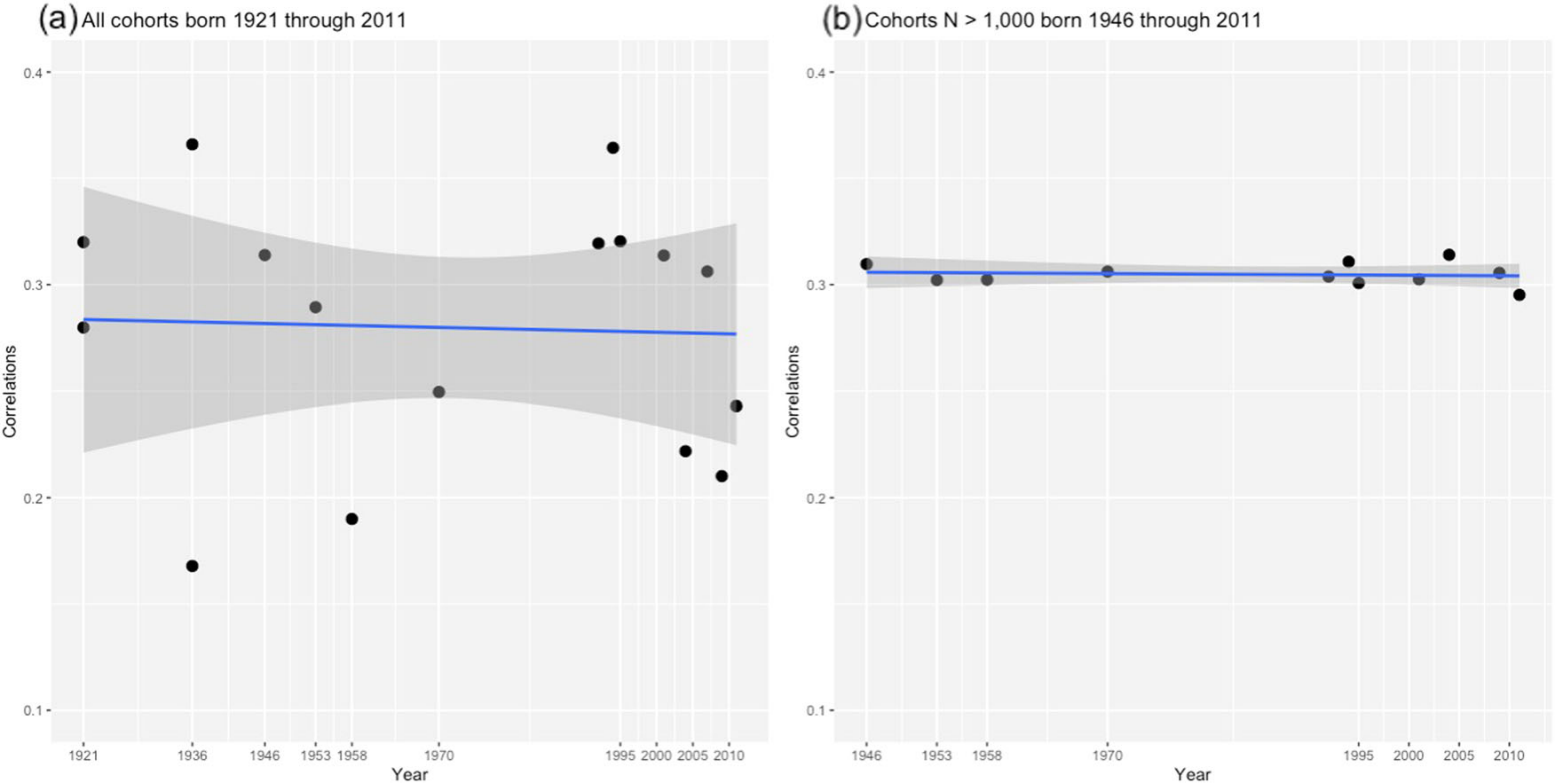
Linear trends in the association between family SES and children’s school performance. Note. Estimates are based on the model fitted to all cohorts (**a**) and the model fitted to cohorts with *N* > 1000 only (**b**).

### Robustness analyses

To test the robustness of our results, we repeated our analyses in those cohorts with more than 1000 participants (*k* = 11, *N* = 89,552), who were born between 1946 and 2011. After adjusting for cohort-specific confounders, family SES correlated on average 0.30 [0.25–0.36] with children’s school performance (Fig. 2, panel (b); full model details are in the SI). The associations varied minimally from 0.30 to 0.31 across cohorts. Figure. 3 shows the slopes mapped onto the cohorts’ estimates, mirroring the results displayed in the forest plots (Fig. 2) and illustrating the stability of the association between family SES and children’s school performance. Finally, we also tested the association between family SES and school performance in the 5 cohorts (overall *N* = 50,306) that were designed to be representative of the wider UK population at their inception (see SI for details). An unadjusted model produced an estimate of 0.33 [0.22–0.43], while a model that included all cohort-specific confounders was nonidentified (i.e., no degrees of freedom, because of the small number of UK population-representative cohorts). Differences in the estimates’ effect sizes between the analyses including cohorts with *N* > 1000 versus those with a UK representative population are likely to be due to controlling for confounders in the former but not in the latter.

### The 90/10 percentile method

**Fig. 4:**
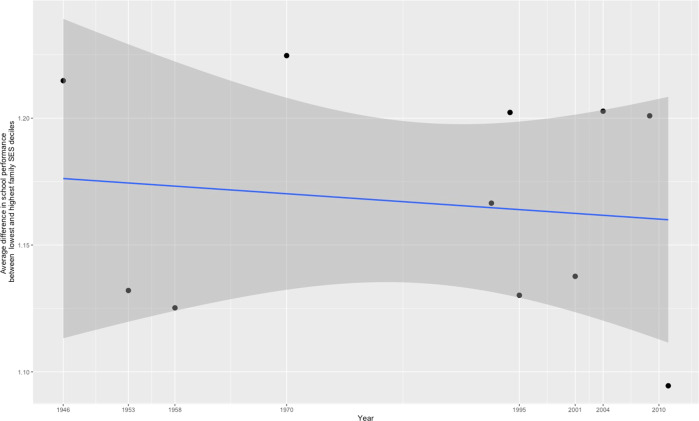
Changes in the school performance gap between the highest and lowest family SES deciles from 1946 through 2011. Note. Points reflect distance in standardized school performance between highest and lowest SES decile, after adjusting for cohort-specific characteristics. The line marks the linear regression; grey areas reflect Confidence Intervals of 95%.

To supplement our correlation-based results, we applied the 90/10 percentile method, which involves estimating the school performance gap between children in the lowest and highest SES deciles and comparing the gaps’ magnitude across cohorts. This method, which is typically applied in sociological and economics research^5–7^, requires large sample sizes to reliably compare the populations at the extreme ends of the SES distribution. Figure 4 shows the results from the 90/10 percentile method in samples with more than 1000 participants (*k* = 11, *N* = 89,552), after adjusting for cohort-specific confounders. A linear regression line suggested that a minimal reduction in the gap in children’s school performance between the lowest and highest family SES decile over time. However, this trend was consistently framed by very large, overlapping Confidence Intervals that signal that a true effect is unlikely. Generally, the 90/10 percentile method, which relies on extreme group comparisons, seemed to produce more spread-out estimates than our correlation-based analysis that involves the entire data distribution.

## Discussion

Children from low SES families perform on average worse throughout compulsory schooling than children from privileged family backgrounds, and as a result, they achieve overall fewer educational qualifications and experience less favorable life outcomes. We showed here that the association between family SES and children’s school performance has remained constant across generations over the past 95 years in Britain. This finding suggests the link between family background and children’s education has not been notably disrupted over the past decades.

Our research advances knowledge in four ways. First, we confirm findings from the only previous study that analyzed data from British populations to test the influence of family SES on children’s education across decades. In 90/10 percentile analyses of test scores from large-scale international student assessment programs, like PISA and TIMSS, Chmielewski^5^ observed a global increase in the disparity in educational achievement between students from high and low SES backgrounds over 50 years. However, for Britain, Chmielewski^5^ found the educational achievement gap between children from low and high SES families to be stagnant. We corroborate and extend these findings in our analyses of children’s school performance in 16 population cohorts that span over 95 years.

Second, our analyses revealed a correlation between family SES and children’s school performance across generations of medium effect size, at least by conventional standards of effect size interpretations. This correlation estimate may appear modest, given that family SES is thought to exert pervasive, long-term influence on all important life outcomes, and that it is one of the most widely studied constructs in social science^6,9,20^. Yet, the effect size of our estimate aligns with those reported in two seminal meta-analyses, whose samples were mostly drawn from the United States. White^50^ reported an association of *r* = 0.22 between family SES and academic achievement across 101 studies published before 1980, and Sirin^3^ estimated the association to be *r* = 0.28 across 58 studies published between 1990 and 2000. Sirin^3^ cautioned that the two meta-analytic effect sizes should not be directly compared to infer conclusions about temporal trends, because of the studies’ many methodological differences.

Third, our finding of a stable association between family SES and children’s school performance was robust across two statistical approaches, with one relying on correlational analyses, and the other, known as 90/10 percentile method, on comparisons of extreme groups. Extreme group analyses are more sensitive to biases from small samples, cohort-specific characteristics, and shifts in data distributions than correlation estimates. Accordingly, we found here that estimates derived with the 90/10 percentile method were more spread out than those obtained from the correlational approach, although they converged on average on the same result.

Fourth, our research illustrates the value of population cohort studies for studying the temporal stability of associations between social conditions and cognitive-behavioral outcomes. We capitalized on the rich data resources that are currently available in Britain, where large-scale cohort studies have been frequently conceived and followed-up^51^. Preserving these data resources, protecting their accessibility for researchers, and expanding their capacity by continuing to follow up existing population cohorts and creating new ones are key for studying societal change and, by extension, for identifying its causative factors and conditions.

Despite its many strengths, our study is not without limitations. First, the population cohorts included in our analyses differed in several characteristics other than their year of birth. For example, the assessment ages of school performance ranged from age 5 to 11 years, with some cohorts recording children’s school grades, others using teacher ratings, and again others including standardized achievement tests’ scores. Likewise, the assessment of SES differed across cohorts: some collected repeatedly multiple indicators of family SES (i.e., mothers’ and fathers’ education, occupation, and family income) and others only a couple at one point in time. We argue that these data are nonetheless comparable, because each cohort applied reliable and valid measurements that were appropriate for its respective generation to assess family SES and primary school performance. We also adjusted our models for differences in cohort-specific characteristics (i.e., confounders) to improve their comparability. We acknowledge that residual effects are possible and may bias our results, given the small number of observations (i.e., population cohorts).

Second, some of the cohorts in our analyses sampled comparatively small populations that may afford low or inconsistent statistical power (see SI for a discussion of power across cohorts). To ensure the robustness of findings, we compared the results based on all 16 population cohorts to those from selection of cohorts with *N* > 1,000 each (*k* = 11). We found minimal differences across both analyses, with neither suggesting a systematic increasing or decreasing trend in the association between family SES and children’s school performance over time.

Third, although Britain is rich in population cohort studies, they have been conceived in irregular intervals, resulting in considerable observation gaps of up to 24 years (Fig. 1). Finally, our analyses cannot identify any shifts in the mechanisms that underlie the influence of family SES on children’s school performance. That is, the causes of the association between family SES and children’s school performance may have changed over time, even if the strength of their association did not change itself^52^.

Our findings suggest that in Britain the association between family origin and children’s education has remained stable over time, at least with regard to primary school performance, despite considerable policy efforts to the contrary^32,33^. We speculate that this stability is, at least in part, due to prioritizing the equality of educational opportunity over the equity of education^32,36,53^. Equality of education refers to a system in which all children enjoy the same learning opportunities, for example, they are taught the same curricula at the same pace by the same methods, regardless of their differences in ability, skills, and interests. By contrast, equity in education aims at reducing children’s differences in educational outcomes, by distributing education resources in ways that level the playing field for all children^34,35^ – that is, by tailoring educational provisions to students’ individual characteristics and needs (e.g. ‘personalizing learning’^54^). For example, equity in education can be achieved by allocating more teaching staff to instructing children who are at risk of academic failure and behavioral misconduct, while better-performing children would be looked after by fewer teachers.

Although the idea of equity in education may appeal in principle, its implementation faces two significant challenges. The first is that parents of children who are well prepared for the demands of compulsory schooling are likely to object to the redistribution of education resources that potentially disadvantage their offspring. Because these parents tend to occupy societal positions of power and prestige more often than parents of children who would be the main beneficiaries of equity in education, the appetite for changing the focus of current education policy is likely to be low. Second, equity in education necessarily prescribes inequality in educational opportunity: not all children will receive the same educational provision and attention, but resources will be concentrated on those with the greatest learning needs. Prioritizing individuals in need is common practice in other policy areas where limited resources are allocated to achieve maximum benefits across the population. For example, in healthcare, costly diagnostic methods tend to be reserved for people at the greatest risk for developing a disease, such as mammograms for detecting breast cancer being offered to women aged 50 years and older, rather than to everybody regardless of gender and age. However, tailoring education provisions to children’s individual needs has been traditionally met with great reluctance. We argue that this is at least partly due to education policies having historically introduced—perhaps inadvertently so—systematic disadvantages for subgroups of the population, especially those of low SES^31^. We caution, however, that previous education policies did not seek to redistribute education resources to reduce children’s differences in learning outcomes but to maximize the best students’ educational achievement, for example through ability streaming or tracking^37,38^. Achieving equity in education while preserving the equality of educational opportunity is a difficult balancing act that challenges education policymakers around the world^55^.

Ensuring that all children have equal opportunity to benefit from the compulsory schooling that society affords should be the primary aim of educational policy in putatively meritocratic societies, like Britain. Yet, our findings suggest that children from low SES families have been persistently disadvantaged in education over the past century, arguably because they cannot make use of the learning opportunities that compulsory schooling offers to the same extent as high SES children do. In societies that selectively reward those with educational credentials, redistributing education resources to address and remediate the differential learning needs that children present with, at the start of primary school, is key to weakening the transmission of social and economic inequality across generations.

## Methods

This study was preregistered and data are available here: https://osf.io/a8fwx/. Because data from existing population cohorts were analysed for the current study, it was not necessary to seek ethical approval here. Informed consent was obtained from the participants by the leaders of the respective population cohorts but not from the authors of the current paper.

### Cohorts

We defined the following criteria for cohorts to be included in our analyses: (a) They sample a population representative of Britain, with their geographical scope covering either (i) the UK, (ii) a country within the UK, (iii) a recognized regional unit in the UK, or (iv) a UK city; (b) their sample was born within a defined period (e.g. year or decade), ensuring that the cohort members had been exposed to comparable economic and political conditions; and (c) they included a valid and reliable measure of school performance during the primary school years (i.e. between children’s age 5–11 years), as well as that of at least one of the predefined indicators of family SES (details below) that children experienced before or concurrently with the assessment of their school performance.

We identified cohorts that theoretically met our inclusion criteria (above) through (a) searching for published cohort profiles, (b) searching through online repositories of UK cohort studies (e.g., CLOSER, www.closer.ac.uk), and (c) informal enquiries to UK researchers involved with the development, maintenance, and organization of cohort studies (further details in the search process and overviews of all population cohorts are in the SI).

### Measures

To assess family background, we extracted from each study where available mothers’ and fathers’ (i) education and (ii) occupation, and (iii) family income, from all assessment waves that occurred before or concurrently with the child’s assessment of school performance (see SI for descriptions of all measures). All 16 cohorts recorded mothers’ and/ or fathers’ occupation; for 14 cohorts at least one measure of educational qualification was available; and in 5 cohorts income was assessed. We also extracted measures of children’s school performance that occurred closest in time after the children’s start of primary school. We included scores that stemmed from (i) established, standardized cognitive ability tests (e.g., the British Ability Scales), (ii) teacher ratings of school performance, (iii) exam performance scores, and (iv) parent reports of school performance. We did not consider scores from experimental cognitive measures (e.g., decision-making, single-trial tasks) or from tests that assessed abilities other than cognitive and/ or scholastic ability (e.g., emotional intelligence).

### Analysis strategy

Data access regulations forbid harmonizing and pooling data across the population cohort studies included here. We therefore analyzed each cohort individually and then applied meta-analytic methods to compare findings. In each population cohort study, we (a) recoded inadequate values (e.g., ‘not answered’; ‘vague answer’; ‘other’) as missing values (i.e. N/A); (b) coded variables so that higher values reflected better outcomes (e.g., higher occupational status; higher educational qualifications); and (c) z-transformed all variables. In each population cohort, we excluded participants who did not have at least one score on school performance and data on one SES indicator.

For operationalizing family SES, we applied general concepts of social stratification, rather than trying to adopt time-invariant measures that site families within the stratification structure at the time of the respective population cohort study. Specifically, we built summary indices from all z-transformed SES indicators that were available in a cohort (i.e., adding together mother’s and father’s education, occupation, and the family income where available) and adjusted them for the number of indicators available per child (see the SI for details on missing data). SES is a formative construct that emerges from its indicators; by contrast, reflective traits, for example, ability, are thought to cause their indicators (i.e., a latent trait^13^). Because SES indicators are not required to correlate or share common variance, internal consistency is not relevant for the validity of the cohorts’ respective SES indices^13^.

To capture children’s differences in school performance, some population cohorts included a single measure of school performance and others multiple ones. Where multiple measures were available, we built summary indices from the z-transformed variables that were completed earliest during primary school and adjusted them for the number of measures available per child.

We estimated the correlations between family SES and children’s school performance in each cohort, and we then applied Fisher’s z-transformation to statistically compare the correlations. Next we fitted meta-regression models, using the R package metaphor^56^, to adjust the Fisher’s z-transformed correlation coefficients for cohort-specific characteristics, which we mean-centered, including the type of assessment of school performance; number of available indicators for SES and school performance; age of assessment of SES and school performance; cohorts’ geographical scope; and % of data missing due to attrition, and selective follow-up and data linkage (see the SI for details on missing data).

### Reporting summary

Further information on research design is available in the Nature Research Reporting Summary linked to this article.

## Supplementary information

Reporting Summary

Supplementary Material

## Data Availability

This study was pre-registered and data are available here: https://osf.io/a8fwx/.

## Code availability

The analysis code is available from the corresponding author upon request.
