# Supplementary material for: Persistent association between family socioeconomic status and primary school performance in Britain over 95 years
Source: NPJ Sci Learn. 2022 Apr 20;7:4. doi: 10.1038/s41539-022-00120-3 (PMC9021311; doi:10.1038/s41539-022-00120-3)
Supplement: Supplementary file 1 — Reporting Summary [file 41539_2022_120_MOESM1_ESM.pdf]

nature portfolio

Corresponding author(s): NPJSCILEARN-00484R1

Last updated by author(s): Jan 19, 2022

Reporting Summary

Nature Portfolio wishes to improve the reproducibility of the work that we publish. This form provides structure for consistency and transparency in reporting. For further information on Nature Portfolio policies, see our Editorial Policies and the Editorial Policy Checklist.

Statistics

For all statistical analyses, confirm that the following items are present in the figure legend, table legend, main text, or Methods section.

n/a	Confirmed	
☐	☒	The exact sample size (*n*) for each experimental group/condition, given as a discrete number and unit of measurement
☐	☒	A statement on whether measurements were taken from distinct samples or whether the same sample was measured repeatedly
☐	☒	The statistical test(s) used AND whether they are one- or two-sided<br>*Only common tests should be described solely by name; describe more complex techniques in the Methods section.*
☐	☒	A description of all covariates tested
☐	☒	A description of any assumptions or corrections, such as tests of normality and adjustment for multiple comparisons
☐	☒	A full description of the statistical parameters including central tendency (e.g. means) or other basic estimates (e.g. regression coefficient) AND variation (e.g. standard deviation) or associated estimates of uncertainty (e.g. confidence intervals)
☐	☒	For null hypothesis testing, the test statistic (e.g. *F*, *t*, *r*) with confidence intervals, effect sizes, degrees of freedom and *P* value noted<br>*Give P values as exact values whenever suitable.*
☒	☐	For Bayesian analysis, information on the choice of priors and Markov chain Monte Carlo settings
☐	☒	For hierarchical and complex designs, identification of the appropriate level for tests and full reporting of outcomes
☐	☒	Estimates of effect sizes (e.g. Cohen's *d*, Pearson's *r*), indicating how they were calculated

Our web collection on statistics for biologists contains articles on many of the points above.

Software and code

Policy information about availability of computer code

Data collection	Data came from 16 population cohorts, which are accessible for research purposes and described in detail in the Supplementary Materials.
Data analysis	All analyses were conducted with R, which is a freely available software.

For manuscripts utilizing custom algorithms or software that are central to the research but not yet described in published literature, software must be made available to editors and reviewers. We strongly encourage code deposition in a community repository (e.g. GitHub). See the Nature Portfolio guidelines for submitting code & software for further information.

Data

Policy information about availability of data

All manuscripts must include a data availability statement. This statement should provide the following information, where applicable:

- Accession codes, unique identifiers, or web links for publicly available datasets
- A description of any restrictions on data availability
- For clinical datasets or third party data, please ensure that the statement adheres to our policy

Data are available for researchers via the respective cohorts' study groups and their corresponding websites. Data for this study are available here: https://osf.io/a8fwx/.

Field-specific reporting

Please select the one below that is the best fit for your research. If you are not sure, read the appropriate sections before making your selection.

☐ Life sciences ☒ Behavioural & social sciences ☐ Ecological, evolutionary & environmental sciences

For a reference copy of the document with all sections, see nature.com/documents/nr-reporting-summary-flat.pdf

Behavioural & social sciences study design

All studies must disclose on these points even when the disclosure is negative.

Study description	Quantitative analysis of population cohort studies from the United Kingdom. Observational study design.
Research sample	Overall, 16 population cohort studies from the United Kingdom born between 1921 and 2011. Data from children (primary cohort members) and their parents, as well as from linkage records and teacher reports, were analysed.
Sampling strategy	All
Data collection	We defined the following criteria for cohorts to be included in our analyses: (a) They sample a population representative of Britain, with their geographical scope covering either (i) the UK, (ii) a country within the UK, (iii) a recognized regional unit in the UK, or (iv) a UK city; (b) their sample was born within a defined period (e.g. year or decade), ensuring that the cohort members had been exposed to comparable economic and political conditions; and (c) they included a valid and reliable measure of school performance during the primary school years (i.e. between children's age 5 to 11 years), as well as that of at least one of the predefined indicators of family SES (details below) that children experienced before or concurrently with the assessment of their school performance.
Timing	N/A
Data exclusions	No population cohorts were excluded.
Non-participation	N/A
Randomization	N/A

Reporting for specific materials, systems and methods

We require information from authors about some types of materials, experimental systems and methods used in many studies. Here, indicate whether each material, system or method listed is relevant to your study. If you are not sure if a list item applies to your research, read the appropriate section before selecting a response.

Materials & experimental systems

n/a	Involved in the study	
☒	☐	Antibodies
☒	☐	Eukaryotic cell lines
☒	☐	Palaeontology and archaeology
☒	☐	Animals and other organisms
☐	☒	Human research participants
☒	☐	Clinical data
☒	☐	Dual use research of concern

Methods

n/a	Involved in the study	
☒	☐	ChIP-seq
☒	☐	Flow cytometry
☒	☐	MRI-based neuroimaging

Human research participants

Policy information about studies involving human research participants

Population characteristics	Overall, 16 population cohort studies from the United Kingdom born between 1921 and 2011. Data from children (primary cohort members) and their parents, as well as from linkage records and teacher reports, were analysed.
Recruitment	N/A
Ethics oversight	University of York

Note that full information on the approval of the study protocol must also be provided in the manuscript.
