# Supplementary material for: Persistent association between family socioeconomic status and primary school performance in Britain over 95 years
Source: NPJ Sci Learn. 2022 Apr 20;7:4. doi: 10.1038/s41539-022-00120-3 (PMC9021311; doi:10.1038/s41539-022-00120-3)
Supplement: Supplementary file 2 — Supplementary Material [file 41539_2022_120_MOESM2_ESM.docx]

**Supporting Information**

Persistent association between family socioeconomic status and primary school performance in Britain over 95 years

Contents

[Overview of the identification and selection of population cohort studies 4](#_Toc60402864)

[Table S1: Overview of population cohorts’ characteristics in current analyses 5](#_Toc60402865)

[Table S2: Mixed effects meta-regression model of adjusting Fisher’s z-transformed correlations with cohort-specific confounders as predictors (k = 16) 6](#_Toc60402866)

[Table S3: Mixed effects meta-regression model of adjusting Fisher’s z-transformed correlations with cohort-specific confounders as predictors in population cohorts with N > 1,000 (k = 11) 7](#_Toc60402867)

[Missing Data 8](#_Toc60402868)

[Comments on Statistical Power 9](#_Toc60402869)

[Cohort Descriptions 10](#_Toc60402870)

[Aberdeen Birth Cohort 1921 (Scottish Mental Survey 1932) 10](#_Toc60402871)

[School Performance 10](#_Toc60402872)

[Family SES 10](#_Toc60402873)

[Table S3.1. Descriptives for ABC1921 10](#_Toc60402874)

[Table S3.2. Correlations for ABC1921 10](#_Toc60402875)

[Lothian Birth Cohort 1921 (Scottish Mental Survey 1932) 12](#_Toc60402876)

[School Performance 12](#_Toc60402877)

[Family SES 12](#_Toc60402878)

[Table S4.1. Descriptives for LBC1921 12](#_Toc60402879)

[Table S4.2. Correlations for LBC1921 12](#_Toc60402880)

[Aberdeen Birth Cohort 1936 (Scottish Mental Survey 1947) 13](#_Toc60402881)

[School Performance 13](#_Toc60402882)

[Family SES 13](#_Toc60402883)

[Table S5.1 Descriptives for ABC1936 13](#_Toc60402884)

[Table S5.2. Correlations for ABC1936 13](#_Toc60402885)

[Lothian Birth Cohort 1936 (Scottish Mental Survey 1947) 14](#_Toc60402886)

[School Performance 14](#_Toc60402887)

[Family SES 14](#_Toc60402888)

[Table S6.1 Descriptives for LBC1936 14](#_Toc60402889)

[Table S6.2. Correlations for LBC1936 14](#_Toc60402890)

[National Survey of Health & Development 15](#_Toc60402891)

[School Performance 15](#_Toc60402892)

[Family SES 16](#_Toc60402893)

[Table S7.1. Descriptives for NSHD 18](#_Toc60402894)

[Table S7.2. Correlations for NSHD 19](#_Toc60402895)

[Aberdeen Children of the 1950’s 20](#_Toc60402896)

[School Performance 20](#_Toc60402897)

[Family SES 20](#_Toc60402898)

[Table S8.1. Descriptives for ACONF 22](#_Toc60402899)

[Table S8.2. Correlations for ACONF 22](#_Toc60402900)

[National Child Development Study 1958 23](#_Toc60402901)

[School Performance 23](#_Toc60402902)

[Family SES 23](#_Toc60402903)

[Table S9.1. Descriptives for NCDS 25](#_Toc60402904)

[Table S9.2. Correlations for NCDS 25](#_Toc60402905)

[British Cohort Study 1970 26](#_Toc60402906)

[School Performance 26](#_Toc60402907)

[Family SES 27](#_Toc60402908)

[Table S10.1. Descriptives for BCS 29](#_Toc60402909)

[Table S10.2. Correlations for BCS 30](#_Toc60402910)

[The Avon Longitudinal Study of Parents and Children (ALSPAC) 31](#_Toc60402911)

[School Performance 31](#_Toc60402912)

[Family SES 31](#_Toc60402913)

[Table S11.1. Descriptives for ALSPAC 33](#_Toc60402914)

[Table S11.2. Correlations for ALSPAC 34](#_Toc60402915)

[The Effective Pre-School, Primary and Secondary Education Project (EPPSE) 36](#_Toc60402916)

[School Performance 36](#_Toc60402917)

[Family SES 36](#_Toc60402918)

[Table S12.1. Descriptives for EPPSE 37](#_Toc60402919)

[Table S12.2. Correlations for EPPSE 37](#_Toc60402920)

[Twins Early Development Study (TEDS) 38](#_Toc60402921)

[School Performance 38](#_Toc60402922)

[Family SES 38](#_Toc60402923)

[Table S13.1. Descriptive for TEDS 39](#_Toc60402924)

[Table S13.2. Correlations for TEDS 40](#_Toc60402925)

[Millennium Cohort Study 2000 41](#_Toc60402926)

[School Performance 41](#_Toc60402927)

[Family SES 41](#_Toc60402928)

[Table S14.1. Descriptives for MCS 43](#_Toc60402929)

[Table S14.2. Correlations for MCS 44](#_Toc60402930)

[Growing Up in Scotland (Birth Cohort One) 45](#_Toc60402931)

[School Performance 45](#_Toc60402932)

[Family SES 45](#_Toc60402933)

[Table S15.1. Descriptives for GUSBC1 47](#_Toc60402934)

[Table S15.2. Correlations for GUSBC1 48](#_Toc60402935)

[Wirral Child Health & Development Study 51](#_Toc60402936)

[School Performance 51](#_Toc60402937)

[Family SES 51](#_Toc60402938)

[Table S16.1. Descriptives for WCHADS 53](#_Toc60402939)

[Table S16.2. Correlations for WCHADS 54](#_Toc60402940)

[Born in Bradford 55](#_Toc60402941)

[School Performance 55](#_Toc60402942)

[Family SES 55](#_Toc60402943)

[Table S17.1. Descriptives for BIB 56](#_Toc60402944)

[Table S17.2. Correlations for BIB 56](#_Toc60402945)

[Growing Up in Scotland (Birth Cohort Two) 57](#_Toc60402946)

[School Performance 57](#_Toc60402947)

[Family SES 57](#_Toc60402948)

[Table S18.1. Descriptives for GUSBC2 59](#_Toc60402949)

[Table S18.2 Correlations for GUSBC2 60](#_Toc60402950)

[References 62](#_Toc60402951)

# Overview of the identification and selection of population cohort studies

We defined the following criteria for population cohort studies to be included in our analyses: (a) They sampled a population representative of Britain, with their geographical scope covering either (i) the UK, (ii) a country within the UK, (iii) a recognized regional unit in the UK, or (iv) a UK city; (b) their sample was born within a defined time period (e.g. year or decade), ensuring that the cohort members had been exposed to comparable economic and political conditions; and (c) they included a valid and reliable measure of school performance during the primary school years (i.e. between children's age 5 to 11 years), as well as that of at least one of the predefined indicators of family SES (details below) that children experienced before or concurrently with the assessment of their school performance.

We searched for cohorts that theoretically met our inclusion criteria (above) through (a) screening published cohort profiles, (b) investigating online repositories of UK cohort studies (e.g., CLOSER, www.closer.ac.uk), and (c) informal enquiries to UK researchers involved with the development, maintenance, and organisation of cohort studies. We identified overall 17 cohorts that met our inclusion criteria, and we were able to include 16 of those in our analyses.

The study that we could not include was Understanding Society, which evolved from the British Household Panel Survey and has tracked approximately 40,000 British households since 1991 (https://www.understandingsociety.ac.uk/). This study is not a cohort study, but a panel study, because its participating children were born in different years and decades. For about 4% of the children in this sample (N ≈ 2,000), school performance data are available from the National Pupil Database (NPD). We sought to explore if sufficiently large samples of children could be identified per year within Understanding Society to form ‘cohorts’. Data access, which was granted nine months after submitting an application, is only possible via designated computers, to which we have no longer access due to the Covid-19 pandemic. Our preliminary analyses suggested, however, that it is unlikely that data from Understanding Society will add meaningfully to the current results.

## Table S1: Overview of population cohorts’ characteristics in current analyses

| Cohort acronym | Birth Year | N | %Miss | r | S.E. | n_SES_ | n_School_ | Age_School_ | Age_SES_ | Type | Scope |
| --- | --- | --- | --- | --- | --- | --- | --- | --- | --- | --- | --- |
| ABC1 | 1921 | 240 | 12.73 | 0.092 | 0.064 | 2 | 1 | 11 | 77 | Ability test | City |
| LBC1 | 1921 | 431 | 21.64 | 0.268 | 0.047 | 4 | 1 | 11 | 79 | Ability test | Region |
| LBC2 | 1936 | 914 | 16.22 | 0.174 | 0.033 | 3 | 1 | 11 | 70 | Ability test | Region |
| ABC2 | 1936 | 480 | 3.61 | 0.245 | 0.044 | 2 | 1 | 11 | 64 | Ability test | City |
| NSHD | 1946 | 4,270 | 20.00 | 0.439 | 0.014 | 10 | 4 | 8 | 0.15 | Ability test | UK |
| ACONF | 1953 | 11,288 | 7.10 | 0.411 | 0.009 | 3 | 4 | 11 | 0 | Ability test | City |
| NCDS | 1958 | 14,923 | 14.31 | 0.233 | 0.008 | 4 | 5 | 7 | 0 | School performance | UK |
| BCS | 1970 | 13,033 | 24.22 | 0.285 | 0.008 | 8 | 5 | 5 | 0 | Ability test | UK |
| ALSPAC | 1992 | 8,686 | 38.23 | 0.345 | 0.010 | 17 | 4 | 7 | -0.35 | School performance | Region |
| EPPSE | 1994 | 2,638 | 12.07 | 0.327 | 0.018 | 4 | 2 | 6 | 3 | School performance | Country |
| TEDS | 1995 | 6,223 | 54.77 | 0.299 | 0.012 | 8 | 6 | 7 | 1.50 | School performance | UK |
| MCS | 2001 | 11,857 | 37.00 | 0.322 | 0.009 | 16 | 1 | 5 | 0.75 | School performance | UK |
| GUS1 | 2004 | 1,823 | 65.06 | 0.191 | 0.023 | 25 | 4 | 10 | 0.83 | School performance | Country |
| WCHADS | 2007 | 318 | 75.27 | 0.318 | 0.053 | 8 | 6 | 4.75 | -0.38 | Ability test | Region |
| BIB | 2009 | 10,481 | 24.37 | 0.150 | 0.010 | 3 | 1 | 5 | -0.25 | School performance | City |
| GUS2 | 2011 | 4,330 | 29.33 | 0.225 | 0.015 | 15 | 2 | 5 | 0.83 | Ability test | Country |

Note. %Miss refers to the proportion of data lost to attrition or selective follow-up and data linkeage. Thus, %Miss is the proportion of the sample excluded in estimating a cohort’s correlation between family SES and children’s school performance, relative to the the cohort’s sample size at conception. r refers to the raw correlation (Pearson); S.E. is the Standard Error. n refers to the number of indicators available per cohort for SES and school performance (see subscript). Age_SES_ refers to the age in years at the first assessment of a SES marker; negative values reflect assessments that occurred during pregnancy before birth (i.e. weeks of gestation). Age_School_ refers to the age in years at the assessment of school performance. Type is the cohort’s assessment of school performance, either by standardized ability tests or direct measures of school performance. Scope is the cohort’s geographical sampling scope.

# Table S2: Mixed effects meta-regression model of adjusting Fisher’s z-transformed correlations with cohort-specific confounders as predictors (k = 16)

|  |  |  |  |  |  | CI95% | |
| --- | --- | --- | --- | --- | --- | --- | --- |
|  |  | **Estimate** | **S.E.** | **t.value** | **p** | **lower** | **upper** |
| Intercept |  | 0.277 | 0.024 | 11.364 | <.0001 | 0.217 | 0.336 |
| Type |  | -0.079 | 0.059 | -1.335 | 0.230 | -0.225 | 0.066 |
| n_SES_ |  | -0.004 | 0.008 | -0.505 | 0.632 | -0.023 | 0.015 |
| n_School_ |  | -0.024 | 0.029 | -0.805 | 0.452 | -0.096 | 0.048 |
| Age_School_ |  | 0.032 | 0.020 | 1.588 | 0.163 | -0.018 | 0.082 |
| Age_SES_ |  | -0.005 | 0.002 | -2.168 | 0.073 | -0.011 | 0.001 |
| % Miss |  | 0.000 | 0.003 | -0.121 | 0.907 | -0.007 | 0.006 |
| Scope_Dummy_ | **Region** | 0.163 | 0.102 | 1.608 | 0.159 | -0.085 | 0.412 |
|  | **UK** | 0.138 | 0.101 | 1.371 | 0.220 | -0.109 | 0.386 |
|  | **Country** | 0.043 | 0.099 | 0.431 | 0.682 | -0.200 | 0.286 |

Note. Scope was dummy coded with 4 levels; reference is City. The model accounted for 67.04% of the heterogeneity. All predictors were mean centered prior to being added to the model. For key, see Table S1.

# Table S3: Mixed effects meta-regression model of adjusting Fisher’s z-transformed correlations with cohort-specific confounders as predictors in population cohorts with N > 1,000 (k = 11)

|  |  |  |  |  |  | CI95% | |
| --- | --- | --- | --- | --- | --- | --- | --- |
|  |  | **Estimate** | **S.E.** | **t.value** | **p** | **lower** | **upper** |
| Intercept |  | 0.304 | 0.004 | 74.466 | 0.009 | 0.252 | 0.356 |
| Type |  | -0.156 | 0.018 | -8.642 | 0.073 | -0.385 | 0.073 |
| n_SES_ |  | -0.006 | 0.005 | -1.276 | 0.423 | -0.064 | 0.053 |
| n_School_ |  | -0.046 | 0.012 | -3.839 | 0.162 | -0.197 | 0.105 |
| Age_School_ |  | 0.044 | 0.007 | 6.416 | 0.098 | -0.044 | 0.132 |
| Age_SES_ |  | 0.072 | 0.014 | 5.275 | 0.119 | -0.102 | 0.246 |
| % Miss |  | 0.001 | 0.001 | 0.656 | 0.630 | -0.015 | 0.017 |
| Scope_Dummy_ | **UK** | 0.179 | 0.053 | 3.412 | 0.182 | -0.489 | 0.848 |
|  | **Country** | -0.034 | 0.061 | -0.558 | 0.676 | -0.809 | 0.741 |
|  | **Region** |  |  |  |  |  |  |

Note. Scope was dummy coded with 4 levels; the reference is City. The model accounted for 99.97% of the heterogeneity. All predictors were mean centered prior to being added to the model.

# Missing Data

Missing data in the population cohort studies that were included in the current analyses stemmed from three principal sources. The first was attrition, which is typical in longitudinal studies that follow up individuals (Watson & Wooden, 2009), and accounted for the largest proportion of missing data in most cohorts. Data missing due to attrition are not at random, because attrition selectively affects cohort populations: participants who live in poorer conditions and report less favourable characteristics, for example low SES, weak school performance, and low health, are more likely to be lost to attrition at follow-up (Watson & Wooden, 2009). As a result, cohort populations tend to become less representative of their original sample over time, although most population cohort studies maintain adequate representativeness (e.g. Connelly & Platt, 2014; Elliott & Shepherd, 2006; Power & Elliott, 2006; Rimfeld et al., 2019).

The second source of missing data is due to population cohort studies collecting or linking data only for a subpopulation of the cohorts’ original sample (e.g. Bradshaw, Tipping, Marryat & Corbett, 2007; Sharp et al., 2012; Wadsworth, Kuh, Richards & Hardy, 2006; Wright et al., 2013). For the cohorts in the current analysis, it was not possible to differentiate if data were missing because of attrition or because of selective follow-up and data linkage. Thus, we included both kinds of missing data in one statistic (i.e. %Miss, Table S1). It is unknown how and to what extent selective follow-up and data linkage affect the representativeness of cohort populations studies and thus, if they cause data to be missing at random or not at random.

The third source of missing data is observations that are missing for individuals within a population cohort study. This type of missing data is not due to attrition or selective follow-up or data linkage but typical in all population cohort studies (Watson & Wooden, 2009). Specifically, data have been collected for an individual but only some and not all variables are available for analyses (e.g. missing one of two or more SES indicators). This type of missing data may occur at random, for example due to recording or transcript errors, but it can also be not at random, for example when individuals refuse answering specific questions because of their other characteristics (e.g. low SES individuals being reluctant to report their income).

The population cohort studies included in the current analyses differed in their respective amounts of missing data. Table S1 shows that the proportion of the sample excluded (i.e. %Miss) in estimating a cohort’s correlation between family SES and children’s school performance, relative to the the cohort’s sample size at conception ranged from less than 4% to more than 75% across cohorts. To handle missing data that is due to attrition and selective follow-up or data linkage, we adjusted all our models for the population cohorts’ %Miss. To handle data that were missing for individuals within population cohorts, we adjusted our summary indices of SES and school performance for the number of indicators that were available per child, respectively. Overall, our approach to handling missing data assumes that the three principal sources of missing data have comparable effects on the population cohort studies included in the current analyses.

# Comments on Statistical Power

Sample sizes ranged from 240 to 14,923 across population cohort studies, after excluding cases with missing data. Accordingly, the cohorts vary in their power to detect an effect size for the association between family SES and children's school performance. Correlations stabilize at sample sizes of N = 250 (Schönbrodt & Perugini, 2013) and thus, even the smallest sample included in the current research should produce a fairly reliable estimate. We analysed our power to detect a significant difference between the correlations from the two smallest samples included in this research (i.e. N = 240 and N = 318), which we treat as the lower bound estimate of power for our analyses. Their power to detect a significant difference between correlation values of .1 and .2 was .23, and .25 for a difference from .2 to .3 (see Figure S1 Lower bound). For the largest samples (i.e. upper bound estimate of power; N = 14,923 and N = 13,033), detecting any correlation differences across the range had a power of 1.

Figure S1: Lower and upper power bounds for comparisons of correlation coefficients across cohorts that sampled the smallest and largest populations


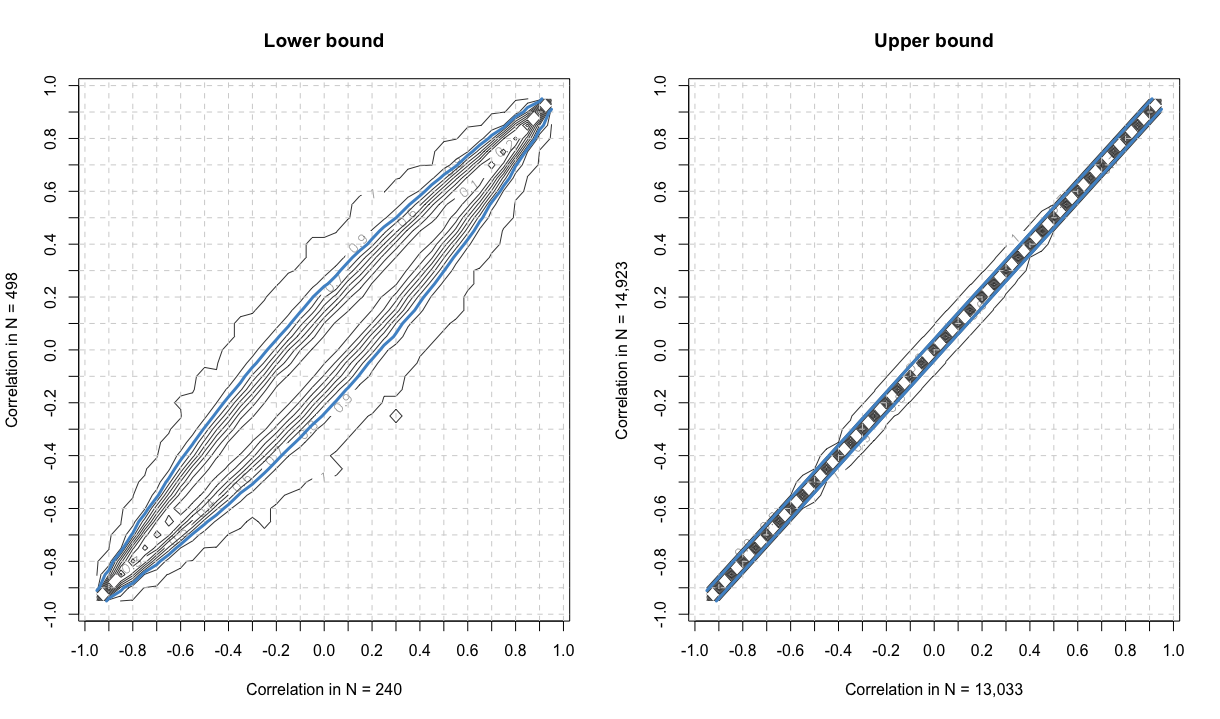


# Cohort Descriptions

We identified overall 16 cohorts, who were born between 1921 and 2011. For each cohort included in the analyses, a brief summary and a description of its respective measures is listed below.

## Aberdeen Birth Cohort 1921 (Scottish Mental Survey 1932)

On the 1^st^ June 1932, 87,498 children who attended school that day in Scotland and were aged between 10.5 and 11.5 years took part in the Scottish Mental Survey (Whalley et al., 2011). The survey, conducted by the Scottish Council for Research in Education, aimed to assess the intelligence of all children born in Scotland 1921. The Aberdeen Birth Cohort 1921 (ABC1921) includes children who completed the Scottish Mental Survey in the city of Aberdeen in 1932 and who were followed up 66 years later (N = 275).

### School Performance

**Sweep 1 (1932).** When the children were around 11 years old, they sat the Moray House Test No. 12 at school, under exam conditions, as part of the Scottish Mental Survey.

*The* *Moray House Test* was devised by Godfrey Thomson, Bell Professor of Education at the University of Edinburgh, and comprised 71 items: following directions (14 items), same–opposites (11 items), word classification (10 items), analogies (8 items), practical (6 items), reasoning (5 items), proverbs (4 items), arithmetic (4 items), spatial (4 items), mixed sentences (3 items), cypher decoding (2 items), and uncategorised (4 items). The maximum score was 76 points. Eight practice items preceded the test.

### Family SES

**Sweep 2 (1998-2001).** At the follow-up at age 77, retrospective demographic information was collected during interviews.

*Occupation.* Fathers’ occupational status was classified as follows: (1) managerial, (2) professional, (3) lesser professional, (4) secretarial, (5) skilled manual, (6) semi-skilled ii, (7) semi-skilled, and (8) unskilled. Mother’s occupational status was scored as: (1) professional administrative, (2) technical, (3) skilled manual, (4) unskilled manual, and (5) unskilled. We recoded the scales, so that lower values indicated lower occupational status.

### Table S3.1. Descriptives for ABC1921

|  | N | M | SD | Med | Min | Max | Skew | Kurtosis | S.E. |
| --- | --- | --- | --- | --- | --- | --- | --- | --- | --- |
| ABC1921 School performance (wave 1) | 354 | 37.62 | 12.95 | 38 | 1 | 69 | -0.41 | -0.13 | 0.69 |
| ABC1921 Father occupation (wave 2) | 237 | 4.04 | 2.20 | 5 | 1 | 8 | 0.02 | -1.20 | 0.14 |
| ABC1921 Mother occupation (wave 2) | 63 | 2.59 | 1.24 | 2 | 1 | 5 | 0.40 | -0.80 | 0.16 |

### Table S3.2. Correlations for ABC1921

|  |  | 1 | 2 | 3 |
| --- | --- | --- | --- | --- |
| 1 | ABC1921 School performance (wave 1) | - |  |  |
| 2 | ABC1921 Father occupation (wave 2) | .10 | - |  |
| 3 | ABC1921 Mother occupation (wave 2) | .23 | .39 | - |

## Lothian Birth Cohort 1921 (Scottish Mental Survey 1932)

The Lothian Birth Cohort 1921 (LBC1921) includes children who completed the Scottish Mental Survey in 1921 in the region of Lothian, and who participated in a follow-up assessment 68 years later at age 79 (N = 550; Taylor, Pattie & Deary, 2018).

### School Performance

**Sweep 1 (1932).** When the children were around 11 years old, they sat the Moray House Test No. 12. For details, see the measure’s description for ABC1921 above.

### Family SES

**Sweep 2 (1999 -2001).** In face-to-face interviews, participants recalled information about their parent’s social demographic background, when they were 11 years old.

*Occupation.* Mothers’ and fathers’ social class was calculated using the General Register Office's Census 1951 Classification of Occupations (HMSO, 1956). Fathers’ occupations were coded into 5 categories: (1) Social Class I, (2) Social Class II, (3) Social Class III, (4) Social Class IV, and (5) Social Class V. Mothers’ occupations were coded into 4 categories: (1) Social Class I & II, (2) Social Class III, (3) Social Class IV, and (4) Social Class V. We recoded the scales, so that lower values indicated lower occupational status. Participants without valid data were coded as missing.

*Education*. Mothers and fathers’ education were assessed as the number of years of formal schooling they completed (i.e. raw scores). Unknown data was coded missing.

### Table S4.1. Descriptives for LBC1921

|  | N | M | SD | Med | Min | Max | Skew | Kurtosis | S.E. |
| --- | --- | --- | --- | --- | --- | --- | --- | --- | --- |
| LBC1921 School performance (wave 1) | 496 | 46.44 | 12.05 | 47 | 4 | 71 | -0.49 | 0.17 | 0.54 |
| LBC1921 Father occupation (wave 2) | 482 | 3.27 | 0.93 | 3 | 1 | 5 | -0.14 | 0.15 | 0.04 |
| LBC1921 Mother occupation (wave 2) | 313 | 2.77 | 0.86 | 3 | 1 | 4 | -0.37 | -.044 | 0.05 |
| LBC1921 Father education (wave 2) | 393 | 10.09 | 2.96 | 9 | 0 | 22 | 1.39 | 2.68 | 0.15 |
| LBC1921 Mother education (wave 2) | 390 | 9.68 | 2.36 | 9 | 0 | 20 | 0.95 | 3.99 | 0.12 |

### Table S4.2. Correlations for LBC1921

|  |  | 1 | 2 | 3 | 4 | 5 |
| --- | --- | --- | --- | --- | --- | --- |
| 1 | LBC1921 School performance (wave 1) | - |  |  |  |  |
| 2 | LBC1921 Father occupation (wave 2) | .25 | - |  |  |  |
| 3 | LBC1921 Mother occupation (wave 2) | .21 | .31 | - |  |  |
| 4 | LBC1921 Father education (wave 2) | .11 | .42 | .24 | - |  |
| 5 | LBC1921 Mother education (wave 2) | .10 | .26 | .35 | .74 | - |

## Aberdeen Birth Cohort 1936 (Scottish Mental Survey 1947)

On 4th June 1947, 70,805 Scottish schoolchildren born in 1936, took part in the Scottish Mental Survey 1947, including the same Moray House Test that was already administered in the Scottish Mental Survey 1932. The Aberdeen Birth Cohort 1936 (ABC1936) includes N = 498, who completed the Scottish Mental Survey 1947 in the city of Aberdeen, and who were followed up 55 years later at age 64 years (Whalley et al., 2011).

### School Performance

**Sweep 1 (1947).** When children were around 11 years old, they sat the Moray House Test No. 12. For details, see the measure’s description for ABC1921 above.

### Family SES

**Sweep 2 (1999-2003).** Assessment interviews were conducted, and retrospective demographic information was collected.

*Occupation.* Fathers’ occupational status was classified as follows: (1) managerial, (2) professional, (3) lesser professional, (4) secretarial, (5) skilled manual, (6) semi-skilled ii, (7) semi-skilled, and (8) unskilled. Mother’s occupational status was scored as: (1) professional, (2) administrative, (3) clerical, (4) technical, (5) skilled manual, (6) unskilled manual, and (7) unskilled. We recoded the scales, so that lower values indicated lower occupational status. Participants without valid data were coded as missing.

### Table S5.1 Descriptives for ABC1936

|  | N | M | SD | Med | Min | Max | Skew | Kurtosis | S.E. |
| --- | --- | --- | --- | --- | --- | --- | --- | --- | --- |
| ABC1936 School performance (wave 1) | 483 | 42.31 | 13.28 | 44 | 1 | 72 | -0.64 | 0.29 | 0.60 |
| ABC1936 Father occupation (wave 2) | 498 | 3.73 | 2.30 | 4 | 1 | 9 | 0.44 | -0.73 | 0.10 |
| ABC1936 Mother occupation (wave 2) | 383 | 2.34 | 1.43 | 2 | 1 | 7 | 1.08 | 0.76 | 0.07 |

### Table S5.2. Correlations for ABC1936

|  |  | 1 | 2 | 3 |
| --- | --- | --- | --- | --- |
| 1 | ABC1936 School performance (wave 1) | - |  |  |
| 2 | ABC1936 Father occupation (wave 2) | 0.19 | - |  |
| 3 | ABC1936 Mother occupation (wave 2) | 0.22 | 0.23 | - |

## Lothian Birth Cohort 1936 (Scottish Mental Survey 1947)

The Lothian Birth Cohort 1936 (LBC1921) includes children who completed the Scottish Mental Survey in 1936 in the region of Lothian and who participated in a follow-up assessment 59 years later at age 70 (N = 1,091; Taylor et al., 2018).

### School Performance

**Sweep 1 (1947).** When the children were around 11 years old, they sat the Moray House Test No. 12. For details, see the measure’s description for ABC1921 above.

### Family SES

**Sweep 2 (2004-2007).** Retrospective demographic information was collected during face to face interviews.

*Occupation.* Fathers’ social class was calculated using the General Register Office's Census 1951 Classification of Occupations (HMSO, 1956). Fathers’ occupations were labelled in five categories: (1) Social Class I, (2) Social Class II, (3) Social Class III, (4) Social Class IV, and (5) Social Class V. We recoded the scores, so that lower values indicated lower status. Participants without valid data were coded as missing.

*Education*. Mothers’ and fathers’ education were assessed as the number of years of formal schooling they completed (i.e. raw scores). Unknown data was coded missing.

### Table S6.1 Descriptives for LBC1936

|  | N | M | SD | Med | Min | Max | Skew | Kurtosis | S.E. |
| --- | --- | --- | --- | --- | --- | --- | --- | --- | --- |
| LBC1936 School performance (wave 1) | 1028 | 49.00 | 11.80 | 50 | 1 | 74 | -0.78 | 0.85 | 0.37 |
| LBC1936 Father occupation (wave 2) | 960 | 3.09 | 0.94 | 3 | 1 | 5 | -0.23 | 0.47 | 0.03 |
| LBC1936 Father education (wave 2) | 826 | 9.96 | 2.24 | 9 | 0 | 23 | 1.83 | 5.11 | 0.08 |
| LBC1936 Mother education (wave 2) | 838 | 9.93 | 2.11 | 9 | 0 | 18 | 1.37 | 3.15 | 0.07 |

### Table S6.2. Correlations for LBC1936

|  |  | 1 | 2 | 3 | 4 |
| --- | --- | --- | --- | --- | --- |
| 1 | LBC1936 School performance (wave 1) | - |  |  |  |
| 2 | LBC1936 Father occupation (wave 2) | .21 | - |  |  |
| 3 | LBC1936 Father education (wave 2) | .07 | .37 | - |  |
| 4 | LBC1936 Mother education (wave 2) | .08 | .31 | .66 | - |

## National Survey of Health & Development

The National Survey of Health & Development (NSHD) sampled all 13,687 babies born in England, Scotland, and Wales, during one week of March 1946. The study aimed to explore why birth rates were low and hoped to inform the design of the NHS. A representative proportion of singleton babies born to married parents were selected for follow up at age 8 (39% of original sample; N = 5,362; Wadsworth et al., 2006).

### School Performance

**Sweep 5 (1954).** The tests administered in the NSHD were specifically developed for the study in collaboration with the National Foundation for Educational Research (NFER) in England and Wales (Wadsworth et al., 2006). When the children were 8 years old, they completed four cognitive ability tests including Picture Intelligence, Mechanical Reading, Word Comprehension, and Sentence Reading. The tests were administered by the children’s class teacher over three separate days of the school week. The teacher administered the test after class, alone, to the target child in the classroom, to ensure a quiet testing environment. Teachers and children recorded the answers in a paper booklet. For the first three tests detailed below, the children were seated so that the appropriate page was in front of them, and the instructions or marking page was in front of the teacher. For the fourth test (e.g. the picture intelligence test), the seating was arranged so the teacher could read the instructions while pointing to pictures in front of the children.

*Picture Intelligence Test*. This test was subdivided into three sections. For each correct answer, children scored 1 point.

*Section One - “The Odd One Out”.* Children were shown five black and white drawings, of which four followed a pattern and the fifth did not. Children drew a line under the odd one out. There were 15 items for this part of the test.

*Section Two - “What Comes Next?”* Children were shown a row of pictures that made up a pattern or story. At the end of the row was a blank space. Children had to identify which image came next by picking out of five options the correct. There were 20 items for this part of the test.

*Section Three - “Matching & Pairing”.* Children were shown a pair of images (e.g. a hand and a glove) and another image (e.g. a hat). They then had to correctly identify from a choice of five images the one that matched the hat (e.g. a head). There were 25 items for this part of the test.

*Mechanical Reading (Part A) & Word Comprehension Test (Part B)*. This test assessed reading ability and word understanding.

*For Part A*, the children read aloud a list of 50 words that increased in difficulty. When the children read incorrectly six consecutive words, the test was paused. If there were any other words on the list that the children had not yet reached but could read, the children were asked to read them aloud. If a child initially read aloud a word incorrectly but then corrected themselves, the teacher awarded a point. The total score was the number of words from the list that the children had read aloud correctly.

*For Part B*, children had to define the 50 words from Part A; an exact definition was not required but a general understanding. If further elaboration was necessary, teachers probed or asked to use the word in a sentence. Teachers read the words aloud, so the children heard the correct pronunciation. The children’s score was the total number of words they could define from the list.

*Sentence Reading Test.* This test consisted of 35 items that showed an incomplete sentence, with a list of five-word options to complete the sentence. The children underlined their response in the test booklet, with correct answers earning a score of 1. If a child was unable to do an item, they’d be encouraged to move onto another until it became apparent that the child could not solve any more items correctly; then the test was terminated.

### Family SES

**Sweep 1 (1946).** Information on family background was collected in interviews with mothers during biannual research visits to the family home, starting when children were aged 8 weeks.

*Occupation.* Fathers’ occupational status was coded in nine levels: (1) professional, (2) employer, (3) own account or farmer, (4) salaried, (5) wage earner non-manual, (6) skilled worker, (7) unskilled worker, (8) agricultural worker, and (9) semi-skilled or unknown skilled. We recoded the scores, using the NS-SEC socio-economic grouping system first introduced in 1951 (Rose & Pevalin, 2001): (1) employer, (2) professional, (3) salaried, (4) skilled worker, (5) semi-skilled or unknown skilled, (6) wage earner non-manual, (7) unskilled worker, (8) own account or farmer and (9) agricultural worker. We reversed the scores, so that lower values indicated lower status. Cases where the father was deceased, not working, or did not provide information were coded as missing.

**Sweep 2 (1948)**

*Occupation.* Mothers’ occupations were classified using five levels: (1) professional, (2) office or shop, (3) factory, (4) domestic, to (5) agriculture. We recoded the scores, so that lower values indicated lower occupational status. Housewives, those not working, who had never worked, who did not share their data, or the survey data had been lost, were coded as missing.

**Sweep 3 (1950)**

*Occupation.* Mothers’ and fathers’ occupational status were assessed as described for sweep 1 and 2.

**Sweep 4 (1952)**

*Occupation.* Fathers’ occupation was coded in 10 levels: (0) foreman, (1) professional, (2) employer, (3) own account or farmer, (4) salaried, (5) wage earner non-manual, (6) skilled worker, (7) unskilled worker, (8) agricultural worker, and (9) semi-skilled or unknown skilled. We reordered the scores using the NS-SEC socio-economic classification system: (1) employer, (2) professional, (3) salaried, (4) skilled worker, (5) semi-skilled or unknown skilled, (6) wage earner non-manual, (7) foreman, (8) unskilled worker, (9) own account or farmer and (10) agricultural worker. We recoded the scores, so that lower values indicated lower status. Mothers’ occupational status were assessed as described for wave 2 and 3.

*Education*. Mothers’ and fathers’ highest educational levels were recorded in 8 classes: (1) primary only, (2) primary and no diploma, (3) primary and technical/communications diploma, (4) primary and professional degree/diploma, (5) secondary only, (6) secondary and no diploma, (7) secondary and technical/communications diploma, and (8) secondary and professional degree/diploma. Unknown cases were coded as missing.

**Sweep 5 (1954)**

*Occupation*. Mothers’ and fathers’ occupational status were assessed as described for sweep 4.

### Table S7.1. Descriptives for NSHD

|  | N | M | SD | Med | Min | Max | Skew | Kurtosis | S.E. |
| --- | --- | --- | --- | --- | --- | --- | --- | --- | --- |
| NSHD Picture intelligence test (wave 5) | 4266 | 40.20 | 9.48 | 42 | 0 | 60 | -0.72 | 0.61 | 0.15 |
| NSHD Mechanical reading test part A (wave 5) | 4259 | 17.02 | 10.28 | 17 | 0 | 49 | 0.32 | -0.68 | 0.16 |
| NSHD Sentence reading test (wave 5) | 4259 | 14.21 | 7.78 | 14 | 0 | 34 | 0.04 | -0.75 | 0.12 |
| NSHD Word comprehension test part B (wave 5) | 4259 | 16.28 | 5.99 | 16 | 0 | 40 | 0.18 | 0.25 | 0.09 |
| NSHD Father occupation (wave 1) | 5319 | 4.88 | 2.03 | 5 | 1 | 9 | -0.30 | -0.57 | 0.03 |
| NSHD Mother occupation (wave 2) | 590 | 3.03 | 1.13 | 3 | 1 | 5 | 0.03 | -0.95 | 0.05 |
| NSHD Father occupation (wave 3) | 4568 | 4.95 | 2.03 | 5 | 1 | 9 | -0.34 | -0.65 | 0.03 |
| NSHD Mother occupation (wave 3) | 1004 | 2.92 | 1.12 | 3 | 1 | 5 | 0.19 | -0.91 | 0.04 |
| NSHD Father occupation (wave 4) | 4365 | 5.76 | 2.37 | 6 | 1 | 10 | -0.58 | -0.61 | 0.04 |
| NSHD Mother occupation (wave 4) | 1232 | 2.91 | 1.12 | 3 | 1 | 5 | 0.26 | -0.96 | 0.03 |
| NSHD Father education (wave 4) | 4285 | 2.53 | 2.29 | 1 | 1 | 8 | 1.26 | 0.12 | 0.04 |
| NSHD Mother education (wave 4) | 4341 | 2.21 | 2.04 | 1 | 1 | 8 | 1.55 | 0.99 | 0.03 |
| NSHD Father occupation (wave 5) | 4109 | 4.80 | 2.37 | 6 | 1 | 10 | -0.60 | -0.61 | 0.04 |
| NSHD Mother occupation (wave 5) | 1463 | 2.94 | 1.13 | 3 | 1 | 5 | 0.19 | -1.02 | 0.03 |

### Table S7.2. Correlations for NSHD

|  |  | 1 | 2 | 3 | 4 | 5 | 6 | 7 | 8 | 9 | 10 | 11 | 12 | 13 |
| --- | --- | --- | --- | --- | --- | --- | --- | --- | --- | --- | --- | --- | --- | --- |
| 1 | NSHD Picture intelligence test (wave 5) | - |  |  |  |  |  |  |  |  |  |  |  |  |
| 2 | NSHD Mechanical reading test part A (wave 5) | .52 | - |  |  |  |  |  |  |  |  |  |  |  |
| 3 | NSHD Sentence reading test (wave 5) | .57 | .87 | - |  |  |  |  |  |  |  |  |  |  |
| 4 | NSHD Word comprehension test part B (wave 5) | .58 | .69 | .68 | - |  |  |  |  |  |  |  |  |  |
| 5 | NSHD Father occupation (wave 1) | .17 | .21 | .22 | .25 | - |  |  |  |  |  |  |  |  |
| 6 | NSHD Mother occupation (wave 2) | .18 | .25 | .26 | .33 | .34 | - |  |  |  |  |  |  |  |
| 7 | NSHD Father occupation (wave 3) | .18 | .22 | .22 | .24 | .79 | .32 | - |  |  |  |  |  |  |
| 8 | NSHD Mother occupation (wave 3) | .17 | .26 | .26 | .30 | .31 | .93 | .31 | - |  |  |  |  |  |
| 9 | NSHD Father occupation (wave 4) | .17 | .21 | .22 | .24 | .71 | .27 | .86 | .27 | - |  |  |  |  |
| 10 | NSHD Mother occupation (wave 4) | .21 | .28 | .27 | .29 | .31 | .82 | .29 | .88 | .29 | - |  |  |  |
| 11 | NSHD Father education (wave 4) | .27 | .33 | .35 | .37 | .40 | .39 | .41 | .35 | .39 | .40 | - |  |  |
| 12 | NSHD Mother education (wave 4) | .24 | .29 | .30 | .35 | .33 | .50 | .31 | .48 | .31 | .49 | .58 | - |  |
| 13 | NSHD Father occupation (wave 5) | .18 | .22 | .22 | .24 | .68 | .27 | .81 | .26 | .88 | .29 | .39 | 30 | - |
| 14 | NSHD Mother occupation (wave 5) | .20 | .29 | .30 | .34 | .32 | .81 | .30 | .84 | .29 | .91 | .41 | .48 | .29 |

## Aberdeen Children of the 1950’s

The Children of the 1950’s cohort (ACONF) is made up of individuals, who were born in the city of Aberdeen in Scotland between 1950 and 1956. Since its creation, the study has explored social and biological influences on health. The cohort included 12,150 children, who were followed up at 10.5 year to 11.5 years (Leon, Lawlor, Clark & Macintyre, 2006).

### School Performance

**Sweep 1 (1962-1964).** Within six months of their 11th birthday, children completed four mental ability tests including the Moray House Verbal Reasoning tests I and II, and an Arithmetic and an English test. Local education authorities provided the test materials and corresponding manuals with scoring and standardization instructions. The tests were administered and scored by primary school teachers. These tests were part of the ‘qualifying’ exam, taken at the end of primary school, which determined the type and stream of secondary school pupils would be selected for.

*The Moray House Verbal Reasoning Tests I and II* comprised overall 30 verbal ability tests, including finding synonyms and antonyms to a given reference word, spelling exercises, finding words to complete sentences, and deductive reasoning problems.

*The Arithmetic Test* comprised two sections. The first 40-item section assessed children's ability to add, subtract, multiply, and divide; these arithmetic problems included up to four figures at a time. The second part comprised 44 worded arithmetic problems which required fractions and understanding of non-metric units.

*The English Test* included a set of 16 different tasks, which mainly assessed text comprehension, grammar and spelling. For example, children were presented with a short text paragraph and subsequently completed questions about its general content and featured characters.

### Family SES

**Sweep 1 (1962-1964).** Information on mother's and father's socioeconomic backgrounds was extracted from hospital records.

*Occupation.* Mothers’ and fathers’ occupational status was categorised using The Registrar General's Classification of Occupations 1950 (HMSO, 1956). Fathers’ occupation at the children’s birth was recorded in seven levels: (1) professional, (2) intermediate/technical, (3) other non-manual, (4) skilled manual occupations requiring an apprenticeship, (5) skilled manual other occupations, (6) semi-skilled manual, and (7) unskilled manual. Fathers, who were unemployed or had died before the child's birth, were treated as missing. Mother’s pre-pregnancy occupational class was recorded on a on a six-point scale ranging from: (1) professional and technical, (2) clerical, (3) distributive, (4) skilled manual, (5) semi-skilled manual, and (6) unskilled manual. Mothers with unknown or missing occupation were coded as missing. We recoded the scores, so that lower values indicated lower occupational status.

**Sweep 2 (1962-1964)** *Occupation*. Fathers’ class occupational status in 1962 was recorded in seven levels: (1) professional, (2) intermediate/technical, (3) other non-manual, (4) skilled manual occupations requiring an apprenticeship, (5) skilled manual other occupations, (6) semi-skilled manual, and (7) unskilled manual. We recoded the scores, so that lower values indicated lower occupational status. Those unemployed, disabled, deceased or unclassifiable were coded as missing.

### Table S8.1. Descriptives for ACONF

|  | N | M | SD | Med | Min | Max | Skew | Kurtosis | S.E. |
| --- | --- | --- | --- | --- | --- | --- | --- | --- | --- |
| ACONF Moray house verbal reasoning test I (wave 2) | 11338 | 99.38 | 14.19 | 99 | 50 | 153 | -0.04 | 0.03 | 0.13 |
| ACONF Moray house verbal reasoning test II (wave 2) | 11323 | 99.42 | 14.26 | 99 | 50 | 142 | 0.03 | -0.15 | 0.13 |
| ACONF Arithmetic test (wave 2) | 9678 | 99.51 | 14.31 | 99 | 53 | 143 | 0.01 | -0.27 | 0.15 |
| ACONF English test (wave 2) | 9678 | 99.55 | 14.43 | 99 | 53 | 144 | 0.07 | -0.20 | 0.15 |
| ACONF Father occupation (wave 1) | 11467 | 3.33 | 1.59 | 3 | 1 | 7 | 0.20 | -0.68 | 0.01 |
| ACONF Mother occupation (wave 1) | 9626 | 3.39 | 1.33 | 3 | 1 | 6 | 0.08 | -0.92 | 0.01 |
| ACONF Father occupation (wave 2) | 11231 | 3.58 | 1.62 | 4 | 1 | 7 | 0.13 | -0.78 | 0.02 |

### Table S8.2. Correlations for ACONF

|  |  | 1 | 2 | 3 | 4 | 5 | 6 |
| --- | --- | --- | --- | --- | --- | --- | --- |
| 1 | ACONF Moray house verbal reasoning test I (wave 2) | - |  |  |  |  |  |
| 2 | ACONF Moray house verbal reasoning test II (wave 2) | .92 | - |  |  |  |  |
| 3 | ACONF Arithmetic test (wave 2) | .84 | .85 | - |  |  |  |
| 4 | ACONF English test (wave 2) | .87 | .87 | .80 | - |  |  |
| 5 | ACONF Father occupation (wave 1) | .33 | .33 | .30 | .32 | - |  |
| 6 | ACONF Mother occupation (wave 1) | .31 | .31 | .27 | .30 | .40 | - |
| 7 | ACONF Father occupation (wave 2) | .34 | .34 | .30 | .32 | .73 | .38 |

## National Child Development Study 1958

The National Child Development Study (NCDS) included 17,415 individuals born in England, Scotland and Wales in a single week of 1958. The study was designed to examine factors associated with stillbirth and mortality in the early stages of infancy (Power & Elliott, 2006).

### School Performance

**Sweep 2 (1965).** Academic attainment data was collected from teachers, using a self-report questionnaire, on five topics including oral ability, awareness of the world, reading, creativity, and number work. Teachers rated the children’s abilities relative to other children of the same age. Level of performance were ranked: (1) well above average, (2) above average, (3) average, (4) below average, and (5) well below average.

### Family SES

**Sweep 1 (1958).** Midwives interviewed mothers postpartum.

*Occupation.* Fathers’ occupations were coded in six categories: (2) Social Class I, (3) Social Class II, (4) Social Class III Non-Manual, (5) Social Class III Manual, (6) Social Class IV, and (7) Social Class V. We recoded the scores, so that lower values indicated lower occupational status. Cases with unemployment, sickness, those deceased, retired, classed as students, or those without a husband, were coded as missing.

Mothers’ occupational status before pregnancy was recorded in 19 levels, including (1) teachers, (2) qualified nurse, (3) bank clerks etc., (4) shopkeeper etc., (5) others in social class I or II, (6) nurses not qualified, (7) clerks or typists, (8) shop assistant or hairdressers, (9) garment workers, (10) textile workers skilled, (11) personal services, (12) others in social class III, (13) machinists, (14) textile workers social class IV, (15) personal social class IV, (16) others in social class IV, (17) textile labourer, (18) personal social class V, (19) others in social class V. We recoded these 19 levels into four social class levels, grouping professions according to their skill level and the previous social class coding, resulting in Social Class I/Qualified (1-5), Social Class II/Skilled (6-12), Social Class III/Skilled Manual (13-16), and Social Class IV/Non-Skilled (17-19). We reversed the scores, so that lower values indicated lower occupational status. Cases with unknown data were coded as missing.

**Sweep 2 (1965).** Health visitors conducted face-to-face interviews with the children’s mother at the family home. If the mother was not present, another adult present in the household, often the father, was interviewed.

*Occupation.* Fathers’ occupations were recorded in seven levels: (2) Social Class I, (3) Social Class II, (4) Social Class III Non-Manual, (5) Social Class III Manual, (6) Social Class IV Non-Manual, (7) Social Class IV Manual, and (8) Social Class V. We recoded the scores, so that lower values indicated lower occupational status. Cases with no father figure or unknown data were coded as missing.

*Education*. Information as to whether fathers had stayed at school, past the minimum age, was available for a proportion of individuals. This was coded as: (2) yes had stayed past the minimum school age, and (3) has not stayed past the minimum school age. For our analysis, we combined both variables into one with two levels, including (0) left school at minimum school age and (1) stayed past minimum school age. Those with unknown data were coded as missing.

### Table S9.1. Descriptives for NCDS

|  | N | M | SD | Med | Min | Max | Skew | Kurtosis | S.E. |
| --- | --- | --- | --- | --- | --- | --- | --- | --- | --- |
| NCDS Oral ability teacher rating (wave 2) | 15013 | 3.11 | 0.96 | 3 | 1 | 5 | 0.27 | 0.08 | 0.01 |
| NCDS Awareness of world teacher rating (wave 2) | 15004 | 2.93 | 0.85 | 3 | 1 | 5 | -0.02 | 0 | 0.01 |
| NCDS Reading teacher rating (wave 2) | 14989 | 3.08 | 0.92 | 3 | 1 | 5 | 0.11 | -0.3 | 0.01 |
| NCDS Creative teacher rating (wave 2) | 14997 | 1.85 | 0.83 | 2 | 0 | 4 | 0.19 | 0.01 | 0.01 |
| NCDS Number work teacher rating (wave 2) | 15009 | 2.85 | 0.87 | 3 | 1 | 5 | 0.23 | -0.16 | 0.01 |
| NCDS Father occupation (wave 1) | 16458 | 3.17 | 1.23 | 3 | 1 | 6 | 0.36 | 0 | 0.01 |
| NCDS Mother occupation (wave 1) | 6681 | 2.71 | 0.76 | 3 | 1 | 4 | -0.43 | -0.02 | 0.01 |
| NCDS Father occupation (wave 2) | 14114 | 4.02 | 1.56 | 4 | 1 | 7 | -0.13 | -0.49 | 0.01 |
| NCDS Father education (wave 2) | 14051 | 0.23 | 0.42 | 0 | 0 | 1 | 1.26 | -0.41 | 0.00 |

### Table S9.2. Correlations for NCDS

|  |  | 1 | 2 | 3 | 4 | 5 | 6 | 7 | 8 |
| --- | --- | --- | --- | --- | --- | --- | --- | --- | --- |
| 1 | NCDS Oral ability teacher rating (wave 2) | - |  |  |  |  |  |  |  |
| 2 | NCDS Awareness of world teacher rating (wave 2) | .71 | - |  |  |  |  |  |  |
| 3 | NCDS Reading teacher rating (wave 2) | .64 | .66 | - |  |  |  |  |  |
| 4 | NCDS Creative teacher rating (wave 2) | .57 | .63 | .58 | - |  |  |  |  |
| 5 | NCDS Number work teacher rating (wave 2) | .55 | .61 | .63 | .55 | - |  |  |  |
| 6 | NCDS Father occupation (wave 1) | .26 | .31 | .24 | .22 | .21 | - |  |  |
| 7 | NCDS Mother occupation (wave 1) | .22 | .25 | .21 | .19 | .17 | .31 | - |  |
| 8 | NCDS Father occupation (wave 2) | .27 | .32 | .25 | .22 | .20 | .61 | .30 | - |
| 9 | NCDS Father education (wave 2) | .23 | .28 | .23 | .20 | .18 | .44 | .22 | .43 |

## British Cohort Study 1970

The 1970 British Cohort Study (BCS1970) follows the lives of 17,198 people born in England, Scotland and Wales in a single week of 1970. The study explores an array of factors, from social and educational development to economic circumstances and family upbringing (Elliott & Shepherd, 2006).

### School Performance

**Sweep 2 (1975).** When the study children were 5 years old, trained interviewers administered five cognitive tests at the family home. The tests were administered with breaks, as appropriate for each child. If a child refused to complete a test, the interviewer was advised to attempt another, and return to any refused tests at the end. Answers were recorded within a paper booklet by both the children (tests 1, 2, 4) and interviewer (tests 3, 5).

*The English Picture Vocabulary Test* (Brimer & Dunn 1962) consisted of 56 items. The children were asked to identify out of four options the picture that best represented a word read aloud by the interviewer. Items increased in difficulty and the test was terminated when a child made five consecutive errors. The children received 1 point for each correct answer. Cases not attempted were coded as missing.

*The Complete a Profile Test* (Kalverboer, 1972) asked children to fill in the outline of a human face with features such as the eyes, ears, and nostrils. The test focused on accurate representations. Points were awarded depending on the richness of the drawing produced (e.g. number of eyes, ears and where the mouth was positioned). The maximum score available was 16.

*The Copying Designs Test* (Rutter, Tizard & Whitmore, 1970) assessed children’s ability to reproduce shapes. Children were asked to make two copies of eight different shapes. For all drawings, the nine principles defined in the aforementioned copying design’s test (Pringle, Butler & Davie, 1966) applied. There was no time limit for the task. A score or 0 or 1 was given for each drawing. As not all children completed two copies, a score of 1 was allocated if at least one good copy was made. The total score was calculated by summing the scores for all individual shapes. Where a child did not attempt the test, data was coded as missing.

*The Original Schonell Reading Test* (Schonell, 1971) was adapted for the current study and comprised of 100 words that assessed the reading age of children aged 5 to 14+ years. Mothers reported if their children had already started to read. Only children whose mothers reported that they could read ‘some words’ or ‘some sentences’ took part; the others were awarded a score of 0. A card with 50 words was then shown to the children. Children were asked to read aloud as many of the words as they could. The test was terminated when children made five consecutive mistakes. One point was awarded for each correctly read word.

*The Human Figure Drawing Test* (Goodenough & Harris, 1963) was a modified version of the ‘Draw-a-Man’ test (Goodenough, 1926). Children were asked to ‘make a picture of a man or a lady’. They were asked to draw as best as they could a whole person, not just a face or head. When the drawing was finished, the interviewer asked the children ‘what is it?’, and the children’s responses about different aspects of the drawing were noted. One point was awarded for each item represented in the drawing (e.g. presence of a head, arms, mouth etc.; Koppitz, 1968; Goodenough & Harris, 1963). Children could score a maximum of 30 points.

### Family SES

**Sweep 1 (1970).** Data was collected by midwives in interviews with the mother shortly after birth.

*Occupation*. Fathers’ occupations were coded into six categories: (1) Social Class I, (2) Social Class II, (3) Social Class III Non-Manual, (4) Social Class III Manual, (5) Social Class IV, and (6) Social Class V. Mothers occupational status before pregnancy, was recorded in 5 levels: (1) Social Class I and II, (2) Social Class III Non-Manual, (3) Social Class III Manual, (4) Social Class IV, and (5) Social Class V. We recoded the scores, so that lower values indicated lower occupational status. Housewives and unknown cases were coded as missing.

*Education*. The ages at which mothers and fathers left education were recorded. Ages were given as raw figures.

**Sweep 2 (1975).** Data was available from health visitors’ interviews and paper-based questionnaires completed by the parents.

*Occupation*. Mothers’ and fathers’ social class, based on occupational status, were differentiated into six classes (1 through 5, with social class 3 divided into manual and non-manual). We recoded the scores, so that lower values indicated lower occupational status. Students, volunteers and unknown cases were coded as missing.

*Education*. Mothers’ and fathers’ highest educational qualifications were recorded in six levels:(2) vocational qualification, (3) O-level or equivalent, (4) A-level or equivalent, (5) state registered nurse (SRN), (6) certificate of education, to (7) degree level or equivalent. We recoded the scores, so that lower values indicated lower occupational status. Cases with invalid and missing data were coded as missing.

### Table S10.1. Descriptives for BCS

|  | N | M | SD | Med | Min | Max | Skew | Kurtosis | S.E. |
| --- | --- | --- | --- | --- | --- | --- | --- | --- | --- |
| BCS English picture vocabulary test (wave 2) | 10274 | 32.95 | 9.46 | 34 | 5 | 51 | -0.69 | -0.10 | 0.09 |
| BCS Complete a profile test (wave 2) | 12451 | 6.92 | 3.99 | 8 | 0 | 16 | 0.11 | -1.28 | 0.04 |
| BCS Copying designs test (wave 2) | 13028 | 4.73 | 1.98 | 5 | 0 | 8 | 0.04 | -0.93 | 0.02 |
| BCS Original Schonell reading test (wave 2) | 5659 | 3.34 | 5.38 | 1 | 0 | 50 | 3.67 | 18.96 | 0.07 |
| BCS Human figure drawing test (wave 2) | 12824 | 0.00 | 1.00 | 0.01 | -3.16 | 3.8 | 0.13 | -0.01 | 0.01 |
| BCS Father occupation (wave 1) | 15773 | 3.22 | 1.21 | 3 | 1 | 6 | 0.45 | 0.00 | 0.01 |
| BCS Mother occupation (wave 1) | 10476 | 3.37 | 1.12 | 4 | 1 | 5 | -0.26 | -1.24 | 0.01 |
| BCS Father education (wave 1) | 16172 | 15.95 | 2.30 | 15 | 6 | 38 | 2.59 | 8.57 | 0.02 |
| BCS Mother education (wave 1) | 16929 | 15.70 | 1.68 | 15 | 6 | 31 | 2.21 | 8.31 | 0.01 |
| BCS Father occupation (wave 2) | 12268 | 3.45 | 1.27 | 3 | 1 | 6 | 0.34 | -0.53 | 0.01 |
| BCS Mother occupation (wave 2) | 4836 | 3.07 | 1.33 | 3 | 1 | 6 | 0.05 | -1.28 | 0.02 |
| BCS Father education (wave 2) | 5850 | 3.13 | 1.95 | 2 | 1 | 6 | 0.58 | -1.30 | 0.03 |
| BCS Mother education (wave 2) | 5434 | 2.27 | 1.43 | 2 | 1 | 6 | 1.34 | 0.90 | 0.02 |

### Table S10.2. Correlations for BCS

|  |  | 1 | 2 | 3 | 4 | 5 | 6 | 7 | 8 | 9 | 10 | 11 | 12 |
| --- | --- | --- | --- | --- | --- | --- | --- | --- | --- | --- | --- | --- | --- |
| 1 | BCS English picture vocab test (wave 2) | - |  |  |  |  |  |  |  |  |  |  |  |
| 2 | BCS Profile test (wave 2) | .16 | - |  |  |  |  |  |  |  |  |  |  |
| 3 | BCS Copying designs test (wave 2) | .33 | .19 | - |  |  |  |  |  |  |  |  |  |
| 4 | BCS Schonell correct response (wave 2) | .12 | .08 | .23 | - |  |  |  |  |  |  |  |  |
| 5 | BCS Human figure drawing standardised test (wave 2) | .24 | .24 | .41 | .14 | - |  |  |  |  |  |  |  |
| 6 | BCS Father occupation (wave 1) | .22 | .08 | .22 | .16 | .13 | - |  |  |  |  |  |  |
| 7 | BCS Mother occupation (wave 1) | .22 | .05 | .21 | .15 | .11 | .40 | - |  |  |  |  |  |
| 8 | BCS Father education (wave 1) | .13 | .06 | .18 | .19 | .10 | .51 | .34 | - |  |  |  |  |
| 9 | BCS Mother education (wave 1) | .16 | .05 | .18 | .20 | .09 | .42 | .43 | .61 | - |  |  |  |
| 10 | BCS Father occupation (wave 2) | .22 | .08 | .24 | .17 | .13 | .66 | .37 | .48 | .40 | - |  |  |
| 11 | BCS Mother occupation (wave 2) | .16 | .04 | .15 | .13 | .10 | .33 | .48 | .30 | .40 | .35 | - |  |
| 12 | BCS Father education (wave 2) | .11 | .05 | .16 | .17 | .08 | .50 | .28 | .54 | .39 | .60 | .29 | - |
| 13 | BCS Mother education (wave 2) | .10 | .04 | .15 | .17 | .07 | .34 | .43 | .45 | .71 | .32 | .42 | .38 |

## The Avon Longitudinal Study of Parents and Children (ALSPAC)

The Avon Longitudinal Study of Parents and Children (ALSPAC) recruited pregnant women in the Bristol area of the UK from 1990 to 92. The original ALSPAC sample consisted of 14,541 pregnancies, with 14,062 live births, born between April 1991 and December 1992. The study explored health and development across the lifespan (Boyd et al., 2013).

### School Performance

**Sweep 3 (1998-1999).** When the children were in Year 2, with ages ranging from 6 years and 9 months to 7 years and 8 months), they sat four Key Stage 1 tests that were administered by the class teacher. The tests included Reading, Writing, Spelling and Maths. The tests were scored by the children's teachers according to National Curriculum levels: (1) level 1, (2) level 2C, (3) level 2B, (4) level 2A, and (5) level 3. Level 1 represents achievement below the national expected standard for most 7-year-olds and level 2 represents achievement at the nationally expected standard for 7-year-olds.

### Family SES

**Sweep 1 (1990-1992).** At 18 weeks gestation, the children’s mother completed a self-reported questionnaire.

*Occupation*. Mothers’ and fathers’ occupational status was grouped in seven levels: (1) higher manager and professional, (2) lower manager and professional, (3) intermediate, (4) small employee or self-employed, (5) lower supervisory and technical, (6) semi routine, and (7) routine. We recoded the scores, so that lower values indicated lower occupational status. Cases with missing data, those unemployed or who had never worked were coded as missing.

*Education*. Mothers’ and fathers’ highest educational qualifications were recorded in five levels: (1) Certificate of Secondary Education, (2) Vocational, (3) General Certificate of Secondary Education, (4) A Level, and (5) Degree. Cases with other professional qualifications, no qualifications or unknown data were coded as missing.

**Sweep 2 (1990-1992).** At 32 weeks gestation, the children’s mothers completed a self-reported questionnaire.

*Occupation*. Mothers’ occupational status was assessed as described for sweep 1.

*Education*. Mothers’ and fathers’ highest educational qualifications were assessed as described for sweep 1.

**Sweep 3 (1992-1993).** At 8 months, the children’s mothers completed a self-reported questionnaire.

*Occupation*. Mothers’ and fathers’ occupational status were assessed as described for sweep 1 and 2.

**Sweep 4 (1993-1994).** At 1 year 9 months, the children’s mothers completed a self-reported questionnaire.

*Occupation*. Mothers’ and fathers’ occupational status were assessed as described for sweep 1, 2 and 3.

**Sweep 5 (1994-1995).** At 2 years 9 months, the children’s mothers completed a self-reported questionnaire.

*Occupation*. Mothers’ occupational status was assessed as described for sweep 1, 2, 3 and 4.

*Income*. Weekly household income was recorded in five levels: (1) less than £100, (2) £100-£199, (3) £200-£299, (4) £300-£399, and (5) more than £400.

**Sweep 6 (1995-1996).** At 3 years 11 months, the children’s mothers and fathers completed a self-reported questionnaire.

*Occupation*. Mothers’ occupational status was assessed as described for sweep 1, 2, 3, 4 and 5.

*Income*. Weekly household income was recorded in five levels: (1) less than £100, (2) £100-£199, (3) £200-£299, (4) £300-£399, and (5) more than £400. Two measures were available, one completed by the child’s mother and one by their father. The two variables were coded using the same scale.

**Sweep 7 (1998-1999).** At 7 years 1 month, the children’s mothers completed a self-reported questionnaire.

*Income*. Weekly household income was recorded in five levels: (1) less than £100, (2) £100-£199, (3) £200-£299, (4) £300-£399, and (5) more than £400.

### Table S11.1. Descriptives for ALSPAC

|  | N | M | SD | Med | Min | Max | Skew | Kurtosis | S.E. |
| --- | --- | --- | --- | --- | --- | --- | --- | --- | --- |
| ALSPAC IQ 1 (wave 7) | 10392 | 3.32 | 1.49 | 3 | 0 | 5 | -0.38 | -1.07 | 0.01 |
| ALSPAC IQ 2 (wave 7) | 10391 | 2.66 | 1.24 | 3 | 0 | 5 | -0.06 | -0.37 | 0.01 |
| ALSPAC IQ 3 (wave 7) | 7376 | 3.64 | 0.93 | 3 | 3 | 5 | 0.78 | -1.40 | 0.01 |
| ALSPAC IQ 4 (wave 7) | 10388 | 3.27 | 1.35 | 3 | 0 | 5 | -0.31 | -0.85 | 0.01 |
| ALSPAC Father occupation (wave 1) | 9348 | 4.48 | 1.93 | 5 | 1 | 7 | -0.34 | -1.15 | 0.02 |
| ALSPAC Mother occupation (wave 1) | 10925 | 4.13 | 1.97 | 5 | 1 | 7 | -0.39 | -1.39 | 0.02 |
| ALSPAC Father education (wave 1) | 9452 | 3.01 | 1.32 | 3 | 1 | 5 | -0.18 | -1.02 | 0.01 |
| ALSPAC Mother education (wave 1) | 9747 | 3.15 | 1.41 | 3 | 1 | 5 | -0.32 | -1.18 | 0.01 |
| ALSPAC Mother occupation (wave 2) | 9537 | 4.49 | 1.79 | 5 | 1 | 7 | -0.76 | -0.77 | 0.02 |
| ALSPAC Father education (wave 2) | 11867 | 3.01 | 1.45 | 3 | 1 | 5 | -0.18 | -1.33 | 0.01 |
| ALSPAC Mother education (wave 2) | 12344 | 2.98 | 1.28 | 3 | 1 | 5 | -0.17 | -0.94 | 0.01 |
| ALSPAC Mother occupation (wave 3) | 4703 | 4.30 | 1.98 | 5 | 1 | 7 | -0.53 | -1.27 | 0.03 |
| ALSPAC Father occupation (wave 3) | 6901 | 4.66 | 1.89 | 5 | 1 | 7 | -0.47 | -1.03 | 0.02 |
| ALSPAC Mother occupation (wave 4) | 4872 | 4.27 | 2.01 | 5 | 1 | 7 | -0.46 | -1.34 | 0.03 |
| ALSPAC Father occupation (wave 4) | 5901 | 4.77 | 1.87 | 6 | 1 | 7 | -0.57 | -0.91 | 0.02 |
| ALSPAC Mother occupation (wave 5) | 4124 | 4.24 | 2.03 | 5 | 1 | 7 | -0.40 | -1.43 | 0.03 |
| ALSPAC Household income (wave 5) | 8763 | 3.34 | 1.26 | 3 | 1 | 5 | -0.20 | -0.98 | 0.01 |
| ALSPAC Mother occupation (wave 6) | 4602 | 4.23 | 2.01 | 5 | 1 | 7 | -0.40 | -1.43 | 0.03 |
| ALSPAC Household income v1 (wave 6) | 4862 | 3.85 | 1.09 | 4 | 1 | 5 | -0.56 | -0.63 | 0.02 |
| ALSPAC Household income v2 (wave 6) | 8572 | 3.47 | 1.26 | 4 | 1 | 5 | -0.33 | -0.95 | 0.01 |
| ALSPAC Household income (wave 7) | 3838 | 4.33 | 0.91 | 5 | 1 | 5 | -1.17 | 0.44 | 0.01 |

### Table S11.2. Correlations for ALSPAC

|  |  | 1 | 2 | 3 | 4 | 5 | 6 | 7 | 8 | 9 | 0 | 11 | 12 | 13 | 14 | 15 | 16 | 17 | 18 | 19 | 20 | 21 |
| --- | --- | --- | --- | --- | --- | --- | --- | --- | --- | --- | --- | --- | --- | --- | --- | --- | --- | --- | --- | --- | --- | --- |
| 1 | ALSPAC IQ 1 (wave 7) | - |  |  |  |  |  |  |  |  |  |  |  |  |  |  |  |  |  |  |  |  |
| 2 | ALSPAC IQ 2 (wave 7) | .81 | - |  |  |  |  |  |  |  |  |  |  |  |  |  |  |  |  |  |  |  |
| 3 | ALSPAC IQ 3 (wave 7) | .49 | .57 | - |  |  |  |  |  |  |  |  |  |  |  |  |  |  |  |  |  |  |
| 4 | ALSPAC IQ 4 (wave 7) | .71 | .70 | .39 | - |  |  |  |  |  |  |  |  |  |  |  |  |  |  |  |  |  |
| 5 | ALSPAC Father occupation (wave 1) | .25 | .26 | .13 | .24 | - |  |  |  |  |  |  |  |  |  |  |  |  |  |  |  |  |
| 6 | ALSPAC Mother occupation (wave 1) | .26 | .26 | .09 | .23 | .35 | - |  |  |  |  |  |  |  |  |  |  |  |  |  |  |  |
| 7 | ALSPAC Father education (wave 1) | .31 | .32 | .15 | .30 | .41 | .47 | - |  |  |  |  |  |  |  |  |  |  |  |  |  |  |
| 8 | ALSPAC Mother education (wave 1) | .28 | .30 | .12 | .28 | .53 | .36 | .54 | - |  |  |  |  |  |  |  |  |  |  |  |  |  |
| 9 | ALSPAC Mother occupation (wave 2) | .26 | .26 | .10 | .24 | .35 | .75 | .47 | .35 | - |  |  |  |  |  |  |  |  |  |  |  |  |
| 10 | ALSPAC Father education (wave 2) | .30 | .30 | .12 | .28 | .52 | .35 | .51 | .79 | .35 | - |  |  |  |  |  |  |  |  |  |  |  |
| 11 | ALSPAC Mother education (wave 2) | .33 | .32 | .14 | .30 | .41 | .47 | .82 | .48 | .48 | .56 | - |  |  |  |  |  |  |  |  |  |  |
| 12 | ALSPAC Mother occupation (wave 3) | .23 | .21 | .08 | .21 | .32 | .64 | .50 | .32 | .64 | .33 | .50 | - |  |  |  |  |  |  |  |  |  |
| 13 | ALSPAC Father occupation (wave 3) | .23 | .24 | .12 | .22 | .77 | .33 | .41 | .52 | .34 | .50 | .41 | .31 | - |  |  |  |  |  |  |  |  |
| 14 | ALSPAC Mother occupation (wave 4) | .20 | .20 | .06 | .19 | .29 | .59 | .46 | .33 | .58 | .32 | .48 | .71 | .30 | - |  |  |  |  |  |  |  |
| 15 | ALSPAC Father occupation (wave 4) | .23 | .23 | .10 | .20 | .75 | .33 | .42 | .52 | .33 | .51 | .41 | .29 | .79 | .29 | - |  |  |  |  |  |  |
| 16 | ALSPAC Mother occupation (wave 5) | .22 | .21 | .09 | .18 | .32 | .56 | .48 | .34 | .53 | .34 | .48 | .65 | .33 | .75 | .32 | - |  |  |  |  |  |
| 17 | ALSPAC Household income (wave 5) | .26 | .26 | .08 | .23 | .44 | .38 | .42 | .43 | .38 | .45 | .43 | .39 | .43 | .39 | .44 | .42 | - |  |  |  |  |
| 18 | ALSPAC Mother occupation (wave 6) | .21 | .21 | .08 | .18 | .31 | .55 | .50 | .33 | .54 | .35 | .49 | .66 | .34 | .69 | .32 | .76 | .42 | - |  |  |  |
| 19 | ALSPAC Household income v1 (wave 6) | .23 | .23 | .10 | .21 | .46 | .36 | .40 | .45 | .35 | .42 | .39 | .39 | .46 | .36 | .46 | .40 | .74 | .41 | - |  |  |
| 20 | ALSPAC Household income v2 (wave 6) | .26 | .26 | .08 | .24 | .43 | .37 | .42 | .43 | .37 | .45 | .42 | .38 | .43 | .38 | .43 | .40 | .80 | .42 | .85 | - |  |
| 21 | ALSPAC Household income (wave 7) | .19 | .20 | .11 | .19 | .42 | .32 | .34 | .40 | .32 | .38 | .35 | .32 | .41 | .30 | .41 | .35 | .62 | .34 | .69 | .64 | - |

## The Effective Pre-School, Primary and Secondary Education Project (EPPSE)

The Effective Pre-School, Primary and Secondary Education (EPPSE) study was established in 1997 to study the influence of early years’ education on development. Over 3000 children were recruited from English Local Authorities and tracked from the start of pre-school when aged 3 years through primary and secondary education, until the age of 16 (Taggart, Sylva, Melhuish, Sammons & Siraj, 2015).

### School Performance

**Sweep 3 (estimate 2000/2001).** When the children were in Year 1 (6 years old), the NFER-Nelson Primary Reading Level 1 and the Maths 6 tests were administered by the class teacher (<https://www.nfer.ac.uk/for-schools/products-services/nfer-tests/nfer-reading-tests/>; <https://www.nfer.ac.uk/for-schools/products-services/nfer-tests/sample-materials/key-stage-1-reading-sample-materials/>)

*Year 1 Reading assessment*. Two child-friendly reading papers are available for use in the year 1 spring and summer terms. The standardised reading papers are tiered and reflect the National Curriculum. Paper one requires teacher support and mediation, while paper two consisting of more challenging questions which requires pupils to work more independently. Children may be required to locate, understand and recall key details from a narrative text; make inferences based on what is said and done; and comprehend a whole sentence in order to select the picture that illustrates the meaning.

*Year 1 Maths assessment*. Two maths booklets are available for use in the year 1 spring and summer terms. The child-friendly standardised assessment focuses upon reasoning and arithmetic ability. The papers are tiered, with paper 1 providing support for calculations and paper 2 requiring children to work more independently with some abstract methods. Teachers were required to read the question a minimum of two times and a maximum of three times. Example questions include, “What number is Jim pointing to on the number line?” and “Tim builds a tower using a cuboid and a pyramid. Tick the two shapes Tim uses.”

### Family SES

**Sweep 1 (estimate 1997).** Interviewers visited the children’s homes and conducted face-to-face interviews with their mothers or other primary caregivers.

*Occupation*. Mothers’ and fathers’ occupational status was grouped in six levels: (1) professional non-manual, (2) other professional non-manual, (3) skilled non-manual, (4) skilled manual, (5) semi-skilled, and (6) unskilled. We recoded the scores, so that lower values indicated lower occupational status. Cases with missing data, those unemployed or who had never worked were coded as missing.

*Education*. Mothers’ and fathers’ highest educational qualifications were recorded in five levels: (1) vocational, (2) 16+ academic, (3) 18+ academic, (4) degree or equivalent, and (5) higher degree. Cases with other professional qualifications, no qualifications or unknown data were coded as missing.

### Table S12.1. Descriptives for EPPSE

|  | N | M | SD | Med | Min | Max | Skew | Kurtosis | S.E. |
| --- | --- | --- | --- | --- | --- | --- | --- | --- | --- |
| EPPSE Y1 Reading (NFER) (wave 3) | 2743 | 20.24 | 7.09 | 19 | 0 | 47 | 0.17 | 0.11 | 0.14 |
| EPPSE Y1 Maths (NFER) (wave 3) | 2734 | 18.66 | 5.52 | 20 | 0 | 26 | -0.94 | 0.44 | 0.11 |
| EPPSE Father occupation (wave 1) | 2373 | 3.72 | 1.34 | 3 | 1 | 6 | 0.04 | -0.93 | 0.03 |
| EPPSE Mother occupation (wave 1) | 2746 | 3.64 | 1.30 | 4 | 1 | 6 | -0.34 | -0.82 | 0.02 |
| EPPSE Father education (wave 1) | 1788 | 2.63 | 1.26 | 2 | 1 | 5 | 0.42 | -1.01 | 0.03 |
| EPPSE Mother education (wave 1) | 2329 | 2.42 | 1.13 | 2 | 1 | 5 | 0.73 | -0.39 | 0.02 |

### Table S12.2. Correlations for EPPSE

|  |  | 1 | 2 | 3 | 4 | 5 |
| --- | --- | --- | --- | --- | --- | --- |
| 1 | EPPSE Y1 Reading (NFER) (wave 3) | - |  |  |  |  |
| 2 | EPPSE Y1 Maths (NFER) (wave 3) | .58 | - |  |  |  |
| 3 | EPPSE Father occupation (wave 1) | .25 | .29 | - |  |  |
| 4 | EPPSE Mother occupation (wave 1) | .27 | .27 | .52 | - |  |
| 5 | EPPSE Father education (wave 1) | .22 | .19 | .59 | .42 | - |
| 6 | EPPSE Mother education (wave 1) | .21 | .23 | .51 | .48 | .61 |

## Twins Early Development Study (TEDS)

Parents of all twins born in England and Wales between 1994 and 1996 were contacted to take part in TEDS. Over 15,000 pairs of twins signed up initially, and approximately 10,000 twins continue to be actively involved until today (Rimfeld et al., 2019). TEDS’ explores how our genetics and the environment influences cognition, learning and behaviour (Oliver & Plomin, 2007). For the current analyses, only data from one randomly selected twin per pair was analysed (N = 13,759).

### School Performance

**Sweep 2 (estimate 2000).** Teachers rated children’s performance in English (i.e. speaking and listening; reading; writing) and Maths (using and applying; numbers; shapes, space and measures) according to National Curriculum levels from (W) working towards level 1, (1) level 1, (2) level 2, (3) level 3, (4+) level 4 or higher. Level 1 and W represent achievement below the national expected standard for most 7 year olds. Level 2 represents achievement at the nationally expected standard for 7 year olds. Level 3 and 4+ represents achievement above the nationally expected standard for most 7 year olds. Level 4 represents the expected level of attainment for pupils aged 11 years.

### Family SES

**Sweep 1 (1994-1996).** A booklet was completed by the twin’s parent or guardian. In the majority of cases, the main respondent was the children’s mother.

*Occupation*. Mothers’ and fathers’ occupational status was grouped in nine levels: (1) manager, (2) professional, (3) technical, (4) clerical, (5) craft, (6) personal, (7) sales, (8) plant, and (9) unskilled. Coding categories were developed from the Standard Occupational Classification 2000 guide (SOC, 2000). We recoded the scores, so that lower values indicated lower occupational status. Cases with unknown or not applicable data were coded as missing.

*Education*. Mothers’ and fathers’ highest educational qualifications were assessed as follows: (2) CSE grade 2-5 or O-level/GCSE grade D-G, (3) CSE grade 1 or O-level/GCSE grade A-C, (4) A-level or S-level, (5) HNC, (6) HND, (7) Undergraduate Degree, and (8) Postgraduate Qualification. Those with no qualifications or unknown data were coded as missing.

**Sweep 2 (estimate 2000).** Postal booklets were sent to the cohort member’s homes. Some parents were also interviewed by telephone; the measures used were identical to those in the booklet.

*Occupation.* Mothers’ and fathers’ occupational status was grouped in nine levels: (1) manager, (2) professional, (3) technical, (4) clerical, (5) craft, (6) personal, (7) sales, (8) plant, and (9) unskilled. We recoded the scores, so that lower values indicated lower occupational status. Cases with unknown or not applicable data were coded as missing.

*Education*. Mothers’ and fathers’ highest academic qualifications were recorded in six classes: (2) 1-4 GCSE’s, (3) 5 or more GCSE’s, (4) A Level, (5) 2 or more A Levels, (6) first degree, and (7) higher degree. Those with no qualifications or other academic qualifications were coded as missing.

### Table S13.1. Descriptive for TEDS

|  | N | M | SD | Med | Min | Max | Skew | Kurtosis | S.E. |
| --- | --- | --- | --- | --- | --- | --- | --- | --- | --- |
| TEDS English speaking & listening teacher rating (wave 2) | 6264 | 2.11 | 0.64 | 2 | 0 | 4 | -0.45 | 1.09 | 0.01 |
| TEDS English reading teacher rating (wave 2) | 6250 | 2.19 | 0.69 | 2 | 0 | 4 | -0.55 | 0.54 | 0.01 |
| TEDS English writing teacher rating (wave 2) | 6222 | 1.96 | 0.65 | 2 | 0 | 4 | -0.66 | 1.58 | 0.01 |
| TEDS Maths using & applying teacher rating (wave 2) | 6252 | 2.05 | 0.65 | 2 | 0 | 4 | -0.52 | 1.10 | 0.01 |
| TEDS Maths numbers teacher rating (wave 2) | 6244 | 2.16 | 0.63 | 2 | 0 | 4 | -0.45 | 0.92 | 0.01 |
| TEDS Maths shapes, space & measurement teacher rating (wave 2) | 6207 | 2.11 | 0.62 | 2 | 0 | 4 | -0.50 | 1.30 | 0.01 |
| TEDS Father occupation (wave 1) | 11087 | 5.99 | 2.60 | 6 | 1 | 9 | -0.39 | -1.10 | 0.02 |
| TEDS Mother occupation (wave 1) | 5751 | 5.90 | 2.26 | 6 | 1 | 9 | -0.46 | -0.70 | 0.03 |
| TEDS Father education (wave 1) | 10361 | 3.37 | 2.03 | 3 | 1 | 7 | 0.53 | -1.15 | 0.02 |
| TEDS Mother education (wave 1) | 12060 | 2.93 | 1.84 | 2 | 1 | 7 | 1.04 | -0.25 | 0.02 |
| TEDS Father occupation (wave 2) | 6392 | 6.45 | 2.47 | 7 | 1 | 9 | -0.77 | -0.51 | 0.03 |
| TEDS Mother occupation (wave 2) | 5444 | 5.65 | 2.25 | 6 | 1 | 9 | -0.45 | -0.62 | 0.03 |
| TEDS Father education (wave 2) | 5141 | 3.15 | 1.89 | 3 | 1 | 6 | 0.18 | -1.57 | 0.03 |
| TEDS Mother education (wave 2) | 6441 | 2.94 | 1.77 | 2 | 1 | 6 | 0.40 | -1.31 | 0.02 |

### Table S13.2. Correlations for TEDS

|  |  | 1 | 2 | 3 | 4 | 5 | 6 | 7 | 8 | 9 | 10 | 11 | 12 | 13 |
| --- | --- | --- | --- | --- | --- | --- | --- | --- | --- | --- | --- | --- | --- | --- |
| 1 | TEDS English speaking & listening teacher rating (wave 2) | - |  |  |  |  |  |  |  |  |  |  |  |  |
| 2 | TEDS English reading teacher rating (wave 2) | .69 | - |  |  |  |  |  |  |  |  |  |  |  |
| 3 | TEDS English writing teacher rating (wave 2) | .66 | .75 | - |  |  |  |  |  |  |  |  |  |  |
| 4 | TEDS Maths using & applying teacher rating (wave 2) | .64 | .68 | .68 | - |  |  |  |  |  |  |  |  |  |
| 5 | TEDS Maths numbers teacher rating (wave 2) | .62 | .68 | .65 | .85 | - |  |  |  |  |  |  |  |  |
| 6 | TEDS Maths shapes, space & measurement teacher rating (wave 2) | .65 | .69 | .68 | .84 | .87 | - |  |  |  |  |  |  |  |
| 7 | TEDS Father occupation (wave 1) | .18 | .19 | .17 | .18 | .18 | .18 | - |  |  |  |  |  |  |
| 8 | TEDS Mother occupation (wave 1) | .13 | .14 | .16 | .16 | .15 | .16 | .31 | - |  |  |  |  |  |
| 9 | TEDS Father education (wave 1) | .20 | .21 | .19 | .20 | .19 | .20 | .46 | .31 | - |  |  |  |  |
| 10 | TEDS Mother education (wave 1) | .23 | .23 | .21 | .22 | .21 | .22 | .33 | .48 | .53 | - |  |  |  |
| 11 | TEDS Father occupation (wave 2) | .19 | .17 | .16 | .16 | .16 | .17 | .57 | .25 | .40 | .28 | - |  |  |
| 12 | TEDS Mother occupation (wave 2) | .16 | .15 | .13 | .14 | .12 | .13 | .24 | .52 | .28 | .43 | .25 | - |  |
| 13 | TEDS Father education (wave 2) | .19 | .20 | .18 | .18 | .17 | .19 | .47 | .32 | .77 | .49 | .42 | .28 | - |
| 14 | TEDS Mother education (wave 2) | .21 | .21 | .19 | .19 | .19 | .19 | .30 | .46 | .47 | .77 | .28 | .43 | .53 |

## Millennium Cohort Study 2000

The Millennium Cohort Study (MCS), also known as ‘Children of the New Century’, recruited 18,818 infants born across England, Scotland, Wales, and Northern Ireland in 2000 to 2001 (Connelly & Platt, 2014). Research topics include, but are not limited to, parenting, relationships and children development.

### School Performance

**Sweep 3 (2006).** *The Early Years Foundation Stage Profile* (EYFSP) is used in England to summarise a children’s attainment at the end of reception. A 16-page teacher survey was designed to mimic the EYFSP to collect data in Wales, Scotland, and Northern Ireland regarding the children’s social, personal, communication, language, literacy and mathematical development. This survey comprised 13 assessment scales, each with nine questions on children’s competencies.

### Family SES

**Sweep 1 (2001).** Researchers visited the children’s’ homes when the children were 9 months old and conducted face-to-face interviews with both resident parents, where appropriate. In the majority of cases, the ‘main’ interview was undertaken by the mother while the ‘partner’ interview was undertaken by the father. Some aspects of the questionnaire were asked to both main and partner respondents; others were asked only to one of them.

*Occupation.* Mothers’ and fathers’ occupational status were grouped in seven levels: (1) higher manager/professional, (2) lower manager/professional, (3) intermediate, (4) small employer/self-employed, (5) lower supervisor/technical, (6) semi routine, and (7) routine. The derived variables were calculated from the full version of the National Statistics Socio-Economic Classification (NS-SEC) containing 42 categories. Classification was assigned by trained coders using answers given to questions about job details. We recoded the scores, so that lower values indicated lower occupational status. Cases with unknown or not applicable data were coded as missing.

*Education*. Mothers’ and fathers’ highest educational qualifications were recorded in six levels: (1) highest degree, (2) first degree, (3) diploma in higher education, (4) A/AS/S level, (5) O level/GCSE grade A-C, (6) GCSE grades D-G. We recoded the scores, so that lower values indicated a lower level qualification. Cases with unknown, refused, and other academic qualifications (not specified) were coded as missing.

*Income*. Annual household income was recorded in six bands: (1) £0 to less than £3,100 per annum (pa), (2) £3,100 to less than £10,400 pa, (3) £10,400 to less than £20,800 pa, (4) £20,800 to less than £31,200, (5) £31,200 to less than £52,000 pa, (6) £52,000 and above pa. Those refused or who only completed a partial interview were coded as missing.

**Sweep 2 (2004).** Interviewers visited the cohort member’s home and conducted face-to-face interviews with both parents.

*Occupation*. Mothers’ and fathers’ occupational status were assessed as described for wave 1.

*Education*. Mothers’ and fathers’ highest academic qualifications were recorded in six levels: (1) highest degree, (2) first degree, (3) diploma in higher education, (4) A/AS/S level, (5) O level/GCSE grade A-C, (6) GCSE grades D-G. We recoded the scores, so that lower values indicated a lower level qualification. Those with overseas qualifications (not specified) or who did not report any, were coded as missing.

*Income*. Annual household income was recorded in six bands: (1) £0 to less than £3,300 pa, (2) £,3300 to less than £11,000 pa, (3) £11,000 to less than £22,000 pa, (4) £22,000 to less than £33,000, (5) £33,000 to less than £55,000 pa, (6) £55,000 and above pa. Cases refused, not applicable or unknown were coded as missing.

**Sweep 3 (2006).** Interviewers visited the cohort members’ home and conducted face-to-face interviews with both parents.

*Occupation*. Mothers’ and fathers’ occupational status were assessed as described for wave 1 & 2.

*Education*. Mothers’ and fathers’ overall highest-level qualifications were coded as: (1) CSE below grade 1, GCSE or O level below grade C, ordinary grades below grade 3, junior certificate below grade C; (2) O Level or GCSE grade A-C, Scottish certificate of education standard, ordinary grades 1-3 or junior certificate grade A-C; (3) AS/A levels, Scottish certificate of education higher, Scottish certificate sixth year studies, leaving certificate or equivalent; (4) first degree, diplomas in higher education, other higher education qualifications, teaching qualifications for schools or further education; and (5) higher degree and postgraduate qualifications, post-graduate diplomas and certificates. Those who did report any were coded missing.

*Income*. Total annual income was available separately for couples and single parents. For couples, income was banded on 19 levels from: (2) Less than £1,600 pa, (3) £1,600 less £3,100 pa, (4) £3,100 less than £4,700 pa, (5) £4,700 less than £6,200 pa, (6) £6,200 less than £7,800 pa, (7) £7,800 less than £10,400 pa, (8) £10,400 less than £13,000 pa, (9) £13,000 less than £15,600 pa, (10) £15,600 less than £18,200 pa, (11) £18,200 less than £20,800 pa, (12) £20,800 less than £26,000 pa, (13) £26,000 less than £31,200 pa, (14) £31,200 less than £36,400 pa, (15) £36,400 less than £41,600 pa, (16) £41,600 less than £46,800 pa, (17) £46,800 less than £52,000 pa, (18) £52,000 less than £80,000 pa, (19) £80,000 less than £100,000 pa, (20) £100,000 or more pa.

For single parents, the categories differed slightly: (2) Less than £1,050 pa, (3) £1,050 less than £2,100 pa, (4) £2,100 less than £3,100 pa, (5) £3,100 less than £4,200 pa, (6) £4,200 less than £5,200 pa, (7) £5,200 less than £7,000 pa, (8) £7,000 less than £8,600 pa, (9) £8,600 less than £10,400 pa, (10) £10,400 less than £12,200 pa, (11) £12,200 less than £13,800 pa, (12) £13,800 less than £17,400 pa, (13) £17,400 less than £20,800 pa, (14) £20,800 less than £24,200 pa, (15) £24,200 less than £27,800 pa, (16) £27,800 less than £31,200 pa, (17) £31,200 less than £34,600 pa, (18) £34,600 less than £52,000 pa, (19) £52,000 less than £66,000 pa, (20) £66,000 or more pa. Cases with not applicable data were coded as missing.

### Table S14.1. Descriptives for MCS

|  | N | M | SD | Med | Min | Max | Skew | Kurtosis | S.E. |
| --- | --- | --- | --- | --- | --- | --- | --- | --- | --- |
| MCS Foundation stage profile combined (wave 3) | 11861 | 88.77 | 19.80 | 92 | 0 | 117 | -0.92 | 0.81 | 0.18 |
| MCS Father occupation (wave 1) | 11458 | 4.29 | 2.07 | 4 | 1 | 7 | -0.16 | -1.35 | 0.02 |
| MCS Mother occupation (wave 1) | 8663 | 4.42 | 1.93 | 5 | 1 | 7 | -0.49 | -1.20 | 0.02 |
| MCS Father education (wave 1) | 10180 | 3.03 | 1.56 | 2 | 1 | 6 | 0.50 | -1.09 | 0.02 |
| MCS Mother education (wave 1) | 14343 | 2.85 | 1.45 | 2 | 1 | 6 | 0.65 | -0.79 | 0.01 |
| MCS Household income (wave 1) | 16941 | 3.34 | 1.20 | 3 | 1 | 6 | 0.47 | -0.57 | 0.01 |
| MCS Father occupation (wave 2) | 9397 | 4.35 | 2.01 | 4 | 1 | 7 | -0.28 | -1.24 | 0.02 |
| MCS Mother occupation (wave 2) | 7836 | 4.40 | 1.89 | 5 | 1 | 7 | -0.50 | -1.18 | 0.02 |
| MCS Father education (wave 2) | 792 | 2.85 | 1.56 | 2 | 1 | 6 | 0.65 | -0.87 | 0.06 |
| MCS Mother education (wave 2) | 471 | 2.75 | 1.51 | 2 | 1 | 6 | 0.74 | -0.69 | 0.07 |
| MCS Household income (wave 2) | 13131 | 3.51 | 1.28 | 3 | 1 | 6 | 0.15 | -0.75 | 0.01 |
| MCS Father occupation (wave 3) | 9439 | 4.43 | 2.00 | 4 | 1 | 7 | -0.31 | -1.20 | 0.02 |
| MCS Mother occupation (wave 3) | 8497 | 4.35 | 1.88 | 5 | 1 | 7 | -0.43 | -1.22 | 0.02 |
| MCS Father education (wave 3) | 9479 | 3.09 | 1.15 | 3 | 1 | 5 | -0.07 | -1.10 | 0.01 |
| MCS Mother education (wave 3) | 12786 | 2.97 | 1.13 | 3 | 1 | 5 | -0.05 | -1.11 | 0.01 |
| MCS Couples household income (wave 3) | 12038 | 9.02 | 5.49 | 10 | 2 | 20 | 0.05 | -1.28 | 0.05 |
| MCS Singles household income (wave 3) | 2958 | 4.26 | 3.97 | 2 | 2 | 20 | 1.73 | 2.09 | 0.07 |

Note. Ns for education at sweep 2 are low, because education data were only collected from mothers and fathers who had not provided the information at sweep 1.

### Table S14.2. Correlations for MCS

|  |  | 1 | 2 | 3 | 4 | 5 | 6 | 7 | 8 | 9 | 10 | 11 | 12 | 13 | 14 | 15 | 16 |
| --- | --- | --- | --- | --- | --- | --- | --- | --- | --- | --- | --- | --- | --- | --- | --- | --- | --- |
| 1 | MCS Foundation stage profile combined (wave 3) | - |  |  |  |  |  |  |  |  |  |  |  |  |  |  |  |
| 2 | MCS Father occupation (wave 1) | .20 | - |  |  |  |  |  |  |  |  |  |  |  |  |  |  |
| 3 | MCS Mother occupation (wave 1) | .21 | .38 | - |  |  |  |  |  |  |  |  |  |  |  |  |  |
| 4 | MCS Father education (wave 1) | .19 | .56 | .36 | - |  |  |  |  |  |  |  |  |  |  |  |  |
| 5 | MCS Mother education (wave 1) | .23 | .40 | .50 | .51 | - |  |  |  |  |  |  |  |  |  |  |  |
| 6 | MCS Household income (wave 1) | .27 | .47 | .48 | .41 | .44 | - |  |  |  |  |  |  |  |  |  |  |
| 7 | MCS Father occupation (wave 2) | .18 | .77 | .35 | .54 | .39 | .45 | - |  |  |  |  |  |  |  |  |  |
| 8 | MCS Mother occupation (wave 2) | .17 | .35 | .77 | .34 | .47 | .44 | .34 | - |  |  |  |  |  |  |  |  |
| 9 | MCS Father education (wave 2) | .21 | .42 | .38 | .43 | .51 | .33 | .53 | .41 | - |  |  |  |  |  |  |  |
| 10 | MCS Mother education (wave 2) | .30 | .52 | .42 | .51 | .65 | .46 | .45 | .37 | .56 | - |  |  |  |  |  |  |
| 11 | MCS Household income (wave 2) | .27 | .46 | .46 | .41 | .44 | .71 | .47 | .45 | .38 | .45 | - |  |  |  |  |  |
| 12 | MCS Father occupation (wave 3) | .18 | .74 | .35 | .52 | .38 | .46 | .89 | .34 | .52 | .46 | .47 | - |  |  |  |  |
| 13 | MCS Mother occupation (wave 3) | .17 | .31 | .70 | .31 | .47 | .41 | .32 | .86 | .40 | .40 | .42 | .31 | - |  |  |  |
| 14 | MCS Father education (wave 3) | .16 | .52 | .31 | .81 | .45 | .37 | .51 | .31 | .89 | .50 | .37 | .52 | .28 | - |  |  |
| 15 | MCS Mother education (wave 3) | .23 | .34 | .47 | .43 | .81 | .39 | .34 | .44 | .48 | .86 | .39 | .33 | .45 | .39 | - |  |
| 16 | MCS Couples household income (wave 3) | .25 | .46 | .42 | .36 | .38 | .54 | .47 | .41 | .33 | .48 | .55 | .52 | .42 | .36 | .36 | - |
| 17 | MCS Singles household income (wave 3) | .17 | .27 | .47 | .26 | .37 | .44 | .26 | .49 | .23 | .30 | .49 | NA | .51 | NA | .33 | NA |

Note. NAs in this table are due to income being recorded either for single- or couple-households. We combined both records into one variable for our analyses.

## Growing Up in Scotland (Birth Cohort One)

Growing up in Scotland (GUSBC1) was established in 2005 to monitor and evaluate children’s family services, with a focus on early years’ policy (Bradshaw et al., 2007). To date, the project has tracked the lives of three cohorts of children. Birth Cohort 1 tracked 5,217 children born in 2004 to 2005; Birth Cohort 2 tracked 6,127 children born in 2010 to 2011, which is described below; and Birth Cohort 3 tracked around 3,000 children born in 2002 to 2003. In the current analyses, Birth Cohort 1 and 2 were included, but neither cognitive nor academic data was available for children at the start of school in Birth Cohort 3, therefore they were not included.

### School Performance

**Sweep 8 (2014-2015).** Teachers assessed children’s academic ability in line with the Curriculum for Excellence (Scottish Executive, 2004) in four categories: listening and talking; reading; writing; numbers and maths. Teachers rated the children’s abilities as follows: (1) early level, (2) first level, (3) second level or (4) third level. Lower numbers refer to lower ability levels.

### Family SES

**Sweep 1 (2005).** Interviewers visited the children’s’ homes to conduct CAPI with their mothers. Where children’s mothers were not available, children’s main carer was interviewed.

*Occupation*. Mothers’ and fathers’ occupational status were categorised using the National Statistics Socio-Economic Classification (NS-SEC). Five categories included: (1) managerial and professional occupations, (2) intermediate occupations, (3) small employers and own account workers, (4) lower supervisory and technical occupations, and (5) semi-routine and routine occupations. We recoded the scores, so that lower values indicated lower occupational status. Those who had never worked, or did not give employment information, were coded as missing.

*Education*. Mothers’ and fathers’ highest educational qualifications were recorded in four levels: (3) GCSE's, Standard Grades, NVQ Level 2 or below, (4) A -Levels, Highers, NVQ Level 3 or equivalent, (5) Higher National Certificate, Higher National Diploma, NVQ Level 4 or equivalent, (6) Degree, NVQ Level 5 or equivalent. We recoded the scores, so that lower values indicated a lower level qualification. Those with no information or other qualifications, were coded as missing.

*Income*. Annual household income before tax was banded in 17 levels: (1) Less than £3,999 pa, (2) £4,000 - £5,999 pa, (3) £6,000 - £7,999 pa, (4) £8,000 - £9,999 pa, (5) £10,000 - £11,999 pa, (6) £12,000 - £14,999 pa, (7) £15,000 - £17,999 pa, (8) £18,000 - £19,999 pa, (9) £20,000 - £22,999 pa, (10) £23,000 - £25,999 pa, (11) £26,000 - £28,999 pa, (12) £29,000 - £31,999 pa, (13) £32,000 - £37,999 pa, (14) £38,000 - £43,999 pa, (15) £44,000 - £49,999 pa, (16) £50,000 - £55,999 pa, and (17) £56,000 or more pa.

**Sweep 2 (2006-2007).** Interviewers conducted CAPI with the same respondent as in sweep 1. An interview for the partner of the main respondent was also included.

*Occupation*. Mothers’ and fathers’ occupational status were assessed as for sweep 1.

*Education*. Mothers’ and fathers’ highest education qualifications were assessed as for sweep 1.

*Income*. Annual total household income before tax was assessed as for sweep 1.

**Sweep 3 (2007-2008).** Interviewers conducted CAPI with the same respondent as sweeps 1 & 2.

*Occupation*. Mothers’ and fathers’ occupational status were assessed as for sweep 1 & 2.

*Education*. Mothers’ and fathers’ highest education qualification were assessed as for sweep 1 & 2.

*Income*. Total household income was assessed as for sweep 1 & 2.

**Sweep 4 (2008-2009).** Interviewers conducted CAPI with the same respondent as in sweeps 1 to 3

*Occupation*. Mothers’ and fathers’ occupational status were assessed as for sweep 1 to 3.

*Education*. Mothers’ and fathers’ highest education qualification were assessed as for sweep 1 to 3.

*Income*. Total household income was assessed as for sweep 1 to 3.

**Sweep 5 (2009-2010).** Interviewers conducted CAPI with the same respondent as in sweeps 1 to 4.

*Occupation*. Mothers’ and fathers’ occupational status were assessed as for sweep 1 to 4.

*Education*. Mothers’ and fathers’ highest education qualification were assessed as for sweep 1 to 4.

*Income*. Total household income was assessed as for sweep 1 to 4.

### Table S15.1. Descriptives for GUSBC1

|  | N | M | SD | Med | Min | Max | Skew | Kurtosis | S.E. |
| --- | --- | --- | --- | --- | --- | --- | --- | --- | --- |
| GUSBC1 Listening & Talking teacher rating (wave 8) | 1821 | 2.92 | 0.29 | 3 | 1 | 4 | -3.39 | 13.34 | 0.01 |
| GUSBC1 Reading teacher rating (wave 8) | 1823 | 2.90 | 0.32 | 3 | 1 | 4 | -2.89 | 9.10 | 0.01 |
| GUSBC1 Writing teacher rating (wave 8) | 1822 | 2.88 | 0.35 | 3 | 1 | 4 | -2.51 | 6.98 | 0.01 |
| GUSBC1 Numeracy teacher rating (wave 8) | 1820 | 2.91 | 0.32 | 3 | 1 | 4 | -2.73 | 9.22 | 0.01 |
| GUSBC1 Father occupation (wave 1) | 4171 | 3.34 | 1.65 | 4 | 1 | 5 | -0.26 | -1.60 | 0.03 |
| GUSBC1 Mother occupation (wave 1) | 4964 | 3.27 | 1.72 | 4 | 1 | 5 | -0.35 | -1.64 | 0.02 |
| GUSBC1 Father education (wave 1) | 3598 | 2.47 | 1.25 | 2 | 1 | 4 | 0.08 | -1.63 | 0.02 |
| GUSBC1 Mother education (wave 1) | 4721 | 2.42 | 1.23 | 2 | 1 | 4 | 0.15 | -1.58 | 0.02 |
| GUSBC1 Household income (wave 1) | 4682 | 9.70 | 4.66 | 10 | 1 | 17 | -0.10 | -1.14 | 0.07 |
| GUSBC1 Father occupation (wave 2) | 3685 | 3.40 | 1.63 | 4 | 1 | 5 | -0.30 | -1.57 | 0.03 |
| GUSBC1 Mother occupation (wave 2) | 4344 | 3.35 | 1.69 | 4 | 1 | 5 | -0.43 | -1.54 | 0.03 |
| GUSBC1 Father education (wave 2) | 3214 | 2.50 | 1.25 | 2 | 1 | 4 | 0.05 | -1.63 | 0.02 |
| GUSBC1 Mother education (wave 2) | 4142 | 2.47 | 1.23 | 2 | 1 | 4 | 0.09 | -1.59 | 0.02 |
| GUSBC1 Household income (wave 2) | 4262 | 10.48 | 4.61 | 11 | 1 | 17 | -0.28 | -1.07 | 0.07 |
| GUSBC1 Father occupation (wave 3) | 3467 | 3.41 | 1.63 | 4 | 1 | 5 | -0.31 | -1.55 | 0.03 |
| GUSBC1 Mother occupation (wave 3) | 4070 | 3.38 | 1.68 | 4 | 1 | 5 | -0.46 | -1.51 | 0.03 |
| GUSBC1 Father education (wave 3) | 3023 | 2.50 | 1.25 | 2 | 1 | 4 | 0.04 | -1.63 | 0.02 |
| GUSBC1 Mother education (wave 3) | 3884 | 2.52 | 1.23 | 2 | 1 | 4 | 0.02 | -1.59 | 0.02 |
| GUSBC1 Household income (wave 3) | 3926 | 11.08 | 4.50 | 12 | 1 | 17 | -0.40 | -0.95 | 0.07 |
| GUSBC1Father occupation (wave 4) | 3310 | 3.43 | 1.62 | 4 | 1 | 5 | -0.33 | -1.54 | 0.03 |
| GUSBC1Mother occupation (wave 4) | 3891 | 3.40 | 1.67 | 4 | 1 | 5 | -0.48 | -1.47 | 0.03 |
| GUSBC1 Father education (wave 4) | 2930 | 2.51 | 1.25 | 2 | 1 | 4 | 0.03 | -1.64 | 0.02 |
| GUSBC1 Mother education (wave 4) | 3717 | 2.55 | 1.22 | 2 | 1 | 4 | -0.02 | -1.59 | 0.02 |
| GUSBC1 Household income (wave 4) | 3793 | 11.50 | 4.37 | 12 | 1 | 17 | -0.50 | -0.81 | 0.07 |
| GUSBC1 Father occupation (wave 5) | 3167 | 3.45 | 1.62 | 4 | 1 | 5 | -0.36 | -1.53 | 0.03 |
| GUSBC1 Mother occupation (wave 5) | 3743 | 3.38 | 1.67 | 4 | 1 | 5 | -0.46 | -1.49 | 0.03 |
| GUSBC1 Father education (wave 5) | 2797 | 2.54 | 1.25 | 2 | 1 | 4 | -0.01 | -1.64 | 0.02 |
| GUSBC1 Mother education (wave 5) | 3568 | 2.58 | 1.22 | 2 | 1 | 4 | -0.06 | -1.58 | 0.02 |
| GUSBC1 Household income (wave 5) | 3607 | 11.65 | 4.39 | 13 | 1 | 17 | -0.51 | -0.84 | 0.07 |

### Table S15.2. Correlations for GUSBC1

|  |  | 1 | 2 | 3 | 4 | 5 | 6 | 7 | 8 | 9 | 10 | 11 | 12 | 13 |
| --- | --- | --- | --- | --- | --- | --- | --- | --- | --- | --- | --- | --- | --- | --- |
| 1 | GUSBC1 Listening & Talking teacher rating (wave 8) | - |  |  |  |  |  |  |  |  |  |  |  |  |
| 2 | GUSBC1 Reading teacher rating (wave 8) | .77 | - |  |  |  |  |  |  |  |  |  |  |  |
| 3 | GUSBC1 Writing teacher rating (wave 8) | .72 | .80 | - |  |  |  |  |  |  |  |  |  |  |
| 4 | GUSBC1 Numeracy teacher rating (wave 8) | .66 | .72 | .67 | - |  |  |  |  |  |  |  |  |  |
| 5 | GUSBC1 Father occupation (wave 1) | .09 | .11 | .11 | .09 | - |  |  |  |  |  |  |  |  |
| 6 | GUSBC1 Mother occupation (wave 1) | .11 | .13 | .12 | .14 | .34 | - |  |  |  |  |  |  |  |
| 7 | GUSBC1 Father education (wave 1) | .10 | .09 | .10 | .06 | .54 | .31 | - |  |  |  |  |  |  |
| 8 | GUSBC1 Mother education (wave 1) | .11 | .14 | .15 | .14 | .34 | .51 | .44 | - |  |  |  |  |  |
| 9 | GUSBC1 Household income (wave 1) | .12 | .13 | .15 | .14 | .44 | .53 | .39 | .46 | - |  |  |  |  |
| 10 | GUSBC1 Father occupation (wave 2) | .09 | .12 | .11 | .11 | .83 | .34 | .54 | .33 | .43 | - |  |  |  |
| 11 | GUSBC1 Mother occupation (wave 2) | .11 | .13 | .12 | .15 | .34 | .91 | .29 | .49 | .51 | .34 | - |  |  |
| 12 | GUSBC1 Father education (wave 2) | .09 | .08 | .09 | .06 | .55 | .29 | 1.00 | .44 | .38 | .53 | .29 | - |  |
| 13 | GUSBC1 Mother education (wave 2) | .12 | .14 | .16 | .14 | .34 | .50 | .44 | 1.00 | .46 | .32 | .49 | .44 | - |
| 14 | GUSBC1 Household income (wave 2) | .14 | .15 | .15 | .14 | .45 | .53 | .40 | .47 | .80 | .46 | .54 | .40 | .46 |
| 15 | GUSBC1 Father occupation (wave 3) | .10 | .13 | .11 | .11 | .81 | .36 | .54 | .34 | .44 | .94 | .35 | .54 | .34 |
| 16 | GUSBC1 Mother occupation (wave 3) | .10 | .12 | .11 | .14 | .34 | .85 | .28 | .50 | .52 | .34 | .92 | .28 | .50 |
| 17 | GUSBC1 Father education (wave 3) | .10 | .09 | .11 | .07 | .56 | .30 | .98 | .43 | .39 | .54 | .29 | .98 | .43 |
| 18 | GUSBC1 Mother education (wave 3) | .11 | .14 | .15 | .13 | .34 | .48 | .44 | .98 | .44 | .32 | .48 | .43 | .98 |
| 19 | GUSBC1 Household income (wave 3) | .13 | .12 | .14 | .14 | .46 | .52 | .41 | .46 | .78 | .46 | .53 | .41 | .45 |
| 20 | GUSBC1 Father occupation (wave 4) | .10 | .13 | .11 | .11 | .80 | .36 | .54 | .34 | .44 | .92 | .36 | .54 | .34 |
| 21 | GUSBC1 Mother occupation (wave 4) | .10 | .12 | .11 | .13 | .34 | .83 | .28 | .50 | .50 | .34 | .88 | .28 | .50 |
| 22 | GUSBC1 Father education (wave 4) | .09 | .09 | .09 | .07 | .55 | .30 | .97 | .43 | .38 | .53 | .30 | .97 | .43 |
| 23 | GUSBC1 Mother education (wave 4) | .12 | .15 | .16 | .13 | .33 | .48 | .43 | .97 | .43 | .32 | .47 | .43 | .97 |
| 24 | GUSBC1 Household income (wave 4) | .14 | .12 | .13 | .11 | .44 | .51 | .39 | .44 | .74 | .43 | .51 | .39 | .44 |
| 25 | GUSBC1 Father occupation (wave 5) | .10 | .11 | .10 | .09 | .78 | .35 | 55 | .34 | .44 | .90 | .35 | .55 | .34 |
| 26 | GUSBC1 Mother occupation (wave 5) | .08 | .10 | .10 | .11 | .34 | .78 | .28 | .48 | .49 | .33 | .83 | .28 | .48 |
| 27 | GUSBC1 Father education (wave 5) | .10 | .09 | .10 | .07 | .56 | .31 | .96 | .43 | .39 | .54 | .31 | .96 | .43 |
| 28 | GUSBC1 Mother education (wave 5) | .12 | .14 | .15 | .12 | .32 | .48 | .42 | .96 | .43 | .31 | .47 | .42 | .96 |
| 29 | GUSBC1 Household income (wave 5) | .16 | .15 | .17 | .13 | .44 | .50 | .39 | .43 | .73 | .43 | .51 | 39 | .43 |

Table S15.2. Correlations for GUSBC1 continued

|  |  | 15 | 16 | 17 | 18 | 19 | 20 | 21 | 22 | 23 | 24 | 25 | 26 | 27 | 28 |
| --- | --- | --- | --- | --- | --- | --- | --- | --- | --- | --- | --- | --- | --- | --- | --- |
| 1 | GUSBC1 Listening & Talking teacher rating (wave 8) |  |  |  |  |  |  |  |  |  |  |  |  |  |  |
| 2 | GUSBC1 Reading teacher rating (wave 8) |  |  |  |  |  |  |  |  |  |  |  |  |  |  |
| 3 | GUSBC1 Writing teacher rating (wave 8) |  |  |  |  |  |  |  |  |  |  |  |  |  |  |
| 4 | GUSBC1 Numeracy teacher rating (wave 8) |  |  |  |  |  |  |  |  |  |  |  |  |  |  |
| 5 | GUSBC1 Father occupation (wave 1) |  |  |  |  |  |  |  |  |  |  |  |  |  |  |
| 6 | GUSBC1 Mother occupation (wave 1) |  |  |  |  |  |  |  |  |  |  |  |  |  |  |
| 7 | GUSBC1 Father education (wave 1) |  |  |  |  |  |  |  |  |  |  |  |  |  |  |
| 8 | GUSBC1 Mother education (wave 1) |  |  |  |  |  |  |  |  |  |  |  |  |  |  |
| 9 | GUSBC1 Household income (wave 1) |  |  |  |  |  |  |  |  |  |  |  |  |  |  |
| 10 | GUSBC1 Father occupation (wave 2) |  |  |  |  |  |  |  |  |  |  |  |  |  |  |
| 11 | GUSBC1 Mother occupation (wave 2) |  |  |  |  |  |  |  |  |  |  |  |  |  |  |
| 12 | GUSBC1 Father education (wave 2) |  |  |  |  |  |  |  |  |  |  |  |  |  |  |
| 13 | GUSBC1 Mother education (wave 2) |  |  |  |  |  |  |  |  |  |  |  |  |  |  |
| 14 | GUSBC1 Household income (wave 2) |  |  |  |  |  |  |  |  |  |  |  |  |  |  |
| 15 | GUSBC1 Father occupation (wave 3) | - |  |  |  |  |  |  |  |  |  |  |  |  |  |
| 16 | GUSBC1 Mother occupation (wave 3) | .35 | - |  |  |  |  |  |  |  |  |  |  |  |  |
| 17 | GUSBC1 Father education (wave 3) | .55 | .29 | - |  |  |  |  |  |  |  |  |  |  |  |
| 18 | GUSBC1 Mother education (wave 3) | .33 | .49 | .43 | - |  |  |  |  |  |  |  |  |  |  |
| 19 | GUSBC1 Household income (wave 3) | .47 | .52 | .41 | .44 | - |  |  |  |  |  |  |  |  |  |
| 20 | GUSBC1 Father occupation (wave 4) | .98 | .35 | .54 | .34 | .48 | - |  |  |  |  |  |  |  |  |
| 21 | GUSBC1 Mother occupation (wave 4) | .35 | .96 | .28 | .49 | .50 | .34 | - |  |  |  |  |  |  |  |
| 22 | GUSBC1 Father education (wave 4) | .54 | .29 | .98 | .43 | .41 | .55 | .29 | - |  |  |  |  |  |  |
| 23 | GUSBC1 Mother education (wave 4) | .33 | .48 | .42 | .99 | .43 | .33 | .49 | .42 | - |  |  |  |  |  |
| 24 | GUSBC1 Household income (wave 4) | .45 | .51 | .39 | .43 | .84 | .45 | .51 | .39 | .43 | - |  |  |  |  |
| 25 | GUSBC1 Father occupation (wave 5) | .95 | .34 | .55 | .34 | .48 | .96 | .33 | .54 | .34 | .46 | - |  |  |  |
| 26 | GUSBC1 Mother occupation (wave 5) | .34 | .89 | .28 | .47 | .50 | .34 | .93 | .29 | .47 | .50 | .34 | - |  |  |
| 27 | GUSBC1 Father education (wave 5) | .55 | .30 | .98 | .43 | .41 | .55 | .29 | .98 | .42 | .38 | .55 | .29 | - |  |
| 28 | GUSBC1 Mother education (wave 5) | .32 | .47 | .41 | .98 | .43 | .33 | .47 | .42 | .99 | .42 | .33 | .47 | .42 | - |
| 29 | GUSBC1 Household income (wave 5) | .44 | .51 | .39 | .42 | .80 | .46 | .50 | .40 | .42 | .83 | .47 | .51 | .40 | .42 |

## Wirral Child Health & Development Study

The Wirral Child Health and Development Study (WCHADS) began in 2006. The project, funded by the Medical Research Council, aimed to explore the development of childhood conduct issues (Sharp et al., 2012; https://www.liverpool.ac.uk/institute-of-life-and-human-sciences/schools-and-departments/department-of-psychological-sciences/research/first-steps/). First time mothers (aged 18 years and above) were approached at their 20-week scan, between March 2007 and December 2008, at the Arrowe Park Hospital in Merseyside. A total of 1,268 mothers, partners and babies were included in the initial sample, and a sub-sample of 341 was followed-up intensively up to age 9 years, including collecting cognitive ability data; this subsample is included in the current study.

### School Performance

**Sweep 3 (2012-2014).** When the children were around 4.75 years old, six tests assessing cognitive ability were completed. The tests were administered in the study’s child development lab by a trained member of the researcher team. Six subscales of the British Ability Scales Version 2 (BASII; Elliott, Smith & McCulloch, 1996/1997) were used: *Verbal Comprehension; Picture Similarities; Naming Vocabulary, Pattern Construction, Early Number Concepts and Copying*. Testing materials included the BAS administration and scoring booklet, early years core booklet 1 & 2, early years record booklet, pencil & eraser, copying paper, ten green plastic tiles, stopwatch, six yellow and black foam squares, nine yellow and black plastic cubes, similarities response cards, a box of toys, and a CAPI tablet for scoring. For details, see the measure’s description for EPPSE above.

### Family SES

**Sweep 1 (2007-2008).** Questionnaires obtaining family background information were given to mothers (and partners if they attended the clinic) to complete separately at their 20-week scan appointment in the antenatal clinic. A research midwife administered the measures. In cases where the partner was not present, the questionnaire was given to the mother to take home with a return envelope.

*Education*. Mothers’ and fathers’ highest educational levels were based on the total number of qualification types they had obtained. A score of 1 was assigned to each of the following qualifications: Certificate of Secondary Education; National Vocational Qualification; General National Vocational Qualification; A-levels; Higher National Qualification; Higher National Diploma; Further Qualifications; Undergraduate Degree; Postgraduate Degree. Scores ranged from 1 to 8. Unknown cases were coded as missing.

*Income*. Annual total household income was banded in eight levels: (1) up to £10,000 pa, (2) £10,000-£20,000 pa, (3) £21,000-£30,000 pa, (4) £31,000-£40,000 pa, (5) £41,000-£50,000 pa, (6) £51,000-£60,000 pa, (7) £61,000-£70,000 pa, (8) £71,000 and above pa. Cases with not applicable data were coded as missing.

**Sweep 2 (2011-2013).** Family background information was collected via postal questionnaires sent to the cohort members’ homes.

*Income*. Annual total household income was collected from mothers and fathers and banded in eight levels: (1) up to £10,000 pa, (2) £10,000-£20,000 pa, (3) £21,000-£30,000 pa, (4) £31,000-£40,000 pa, (5) £41,000-£50,000 pa, (6) £51,000-£60,000 pa, (7) £61,000-£70,000 pa, (8) £71,000 and above pa. Cases with not applicable data were coded as missing.

*Occupation*. Mothers’ and fathers’ occupational status was derived from the eight NS-SEC categories: (1) modern professional occupations, (2) clerical and intermediate occupations, (3) senior manager or administrators, (4) technical and craft occupations, (5) semi routine manual and service occupations, (6) routine manual and service occupations, (7) middle and junior managers, (8) traditional professional occupations. We used the NS-SEC self-code for employees, resulting in four categories where lower values indicated lower occupational status: (1) semi routine manual and service operations; routine manual and service operations; (2) technical and craft operations; (3) clerical and intermediate occupations; and (4) modern professional occupations; senior managers and administrators; middle and junior managers; traditional professional occupations. Those classed as in training or not in work or were coded as missing.

**Sweep 3 (2012-2014).** Mothers in the intensive sample attended the child development laboratory, during which time they completed a background questionnaire.

*Income*. Total household income was assessed as described for sweep 1 and 2.

### Table S16.1. Descriptives for WCHADS

|  | N | M | SD | Med | Min | Max | Skew | Kurtosis | S.E. |
| --- | --- | --- | --- | --- | --- | --- | --- | --- | --- |
| WCHADS BAS verbal comprehension (wave 3) | 317 | 14.10 | 4.56 | 15 | 4 | 27 | 0.31 | -0.24 | 0.26 |
| WCHADS BAS picture similarities (wave 3) | 311 | 14.90 | 3.46 | 15 | 0 | 22 | -0.87 | 1.30 | 0.20 |
| WCHADS BAS naming vocabulary (wave 3) | 320 | 13.74 | 2.96 | 14 | 5 | 23 | -0.12 | 0.23 | 0.17 |
| WCHADS BAS pattern construction (wave 3) | 310 | 11.66 | 5.90 | 11 | 0 | 36 | 1.62 | 3.99 | 0.34 |
| WCHADS BAS early number concepts (wave 3) | 303 | 14.71 | 3.87 | 15 | 2 | 24 | -0.39 | 0.77 | 0.22 |
| WCHADS BAS copying (wave 3) | 304 | 14.33 | 4.82 | 14 | 3 | 27 | -0.05 | -0.08 | 0.28 |
| WCHADS Household income (wave 1) | 1020 | 4.13 | 1.97 | 4 | 1 | 8 | 0.21 | -0.77 | 0.06 |
| WCHADS Mother education (wave 1) | 922 | 1.61 | 0.74 | 1 | 1 | 4 | 1.03 | 0.49 | 0.02 |
| WCHADS Father education (wave 1) | 747 | 1.71 | 0.86 | 1 | 1 | 6 | 1.22 | 1.48 | 0.03 |
| WCHADS Household income mother (wave 2) | 721 | 4.10 | 2.08 | 4 | 1 | 8 | 0.20 | -0.94 | 0.08 |
| WCHADS Household income father (wave 2) | 449 | 4.57 | 1.94 | 4 | 1 | 8 | 0.14 | -0.85 | 0.09 |
| WCHADS Father occupation (wave 2) | 591 | 2.98 | 1.19 | 4 | 1 | 4 | -0.56 | -1.32 | 0.05 |
| WCHADS Mother occupation (wave 2) | 553 | 3.31 | 1.00 | 4 | 1 | 4 | -1.41 | 0.79 | 0.04 |
| WCHADS Household income (wave 3) | 702 | 4.17 | 2.10 | 4 | 1 | 8 | 0.27 | -0.89 | 0.08 |

### Table S16.2. Correlations for WCHADS

|  |  | 1 | 2 | 3 | 4 | 5 | 6 | 7 | 8 | 9 | 10 | 11 | 12 | 13 |
| --- | --- | --- | --- | --- | --- | --- | --- | --- | --- | --- | --- | --- | --- | --- |
| 1 | WCHADS BAS verbal comprehension (wave 3) | - |  |  |  |  |  |  |  |  |  |  |  |  |
| 2 | WCHADS BAS picture similarities (wave 3) | .41 | - |  |  |  |  |  |  |  |  |  |  |  |
| 3 | WCHADS BAS naming vocabulary (wave 3) | .57 | .34 | - |  |  |  |  |  |  |  |  |  |  |
| 4 | WCHADS BAS pattern construction (wave 3) | .25 | .31 | .17 | - |  |  |  |  |  |  |  |  |  |
| 5 | WCHADS BAS early number concepts (wave 3) | .47 | .40 | .45 | .38 | - |  |  |  |  |  |  |  |  |
| 6 | WCHADS BAS copying (wave 3) | .27 | .22 | .28 | .35 | .40 | - |  |  |  |  |  |  |  |
| 7 | WCHADS Household income (wave 1) | .18 | .19 | .22 | .06 | .17 | .04 | - |  |  |  |  |  |  |
| 8 | WCHADS Mother education (wave 1) | .31 | .24 | .30 | .17 | .18 | .16 | .30 | - |  |  |  |  |  |
| 9 | WCHADS Father education (wave 1) | .10 | .04 | .10 | -.04 | .13 | .02 | .25 | .16 | - |  |  |  |  |
| 10 | WCHADS Household income mother (wave 2) | .20 | .20 | .17 | .09 | .24 | .08 | .78 | .25 | .17 | - |  |  |  |
| 11 | WCHADS Household income father (wave 2) | .16 | .21 | .16 | .06 | .22 | .13 | .73 | .26 | .23 | .89 | - |  |  |
| 12 | WCHADS Father occupation (wave 2) | .14 | .17 | .13 | .08 | .12 | .07 | .38 | .21 | .26 | .45 | .45 | - |  |
| 13 | WCHADS Mother occupation (wave 2) | .17 | .13 | .19 | .07 | .22 | .04 | .37 | .26 | .16 | .46 | .47 | .25 | - |
| 14 | WCHADS Household income (wave 3) | .14 | .16 | .15 | .08 | .21 | .11 | .73 | .26 | .25 | .87 | .87 | .44 | .46 |

## Born in Bradford

The Born in Bradford (BIB) study enrolled 13,858 children, born at Bradford Royal Infirmary between March 2007 and December 2010, and their parents to investigate childhood illness, social development and family environments (Wright et al., 2013).

### School Performance

**Sweep 2 (estimate 2012-2015).** When the children were 5 years old, teachers assessed their EYFSP towards the end of reception. Depending on whether the children started school before or after 2012:

*Pre-2013 EYFSP.* This version was used up until the end of the 2011/2012 academic year and included six sections: (1) creative development, (2) knowledge and understanding of the world, (3) physical development, (4) communication language and literature (reading; language; linking sounds; writing), (5) problems solving, reasoning and numbers (labels; shapes; calculating), and (6) personal, social and emotional development (dispositions & attitudes; emotional development; social development). Children could score a maximum of 114 points.

*Post-2013 EYFSP.* This version was used from the start of the 2012/2013 academic year with seven sections: (1) communication and language (listening; attention; understanding), (2) expressive art and design (media and materials; imaginative), (3) literacy (reading; writing), (4) maths (numbers; shapes), (5) physical development (move and handle; health and self-care), (6) personal, social and emotional development (self-confidence and awareness; feelings and behaviour; relationships), and (7) understanding of the world (people; the world; technology). Scores were either (1) emerging, (2) expected, or (3) exceeding. Children could score a maximum of 51 points.

### Family SES

**Baseline Data (2007-2010).** Women were recruited from the Bradford Royal Infirmary at 26-28 weeks gestation and invited to complete an interview and a questionnaire.

*Occupation*. Fathers’ occupational status was derived from 8 NS-SEC categories: (1) modern professional occupations, (2) clerical and intermediate occupations, (3) senior managers and administrators, (4) technical and craft operations, (5) semi routine manual and service operations, (6) routine manual and service operations, (7) middle and junior manages, and (8) traditional professional occupations. We used the NS-SEC self-code for employees, resulting in four categories where lower values indicated lower occupational status: (1) semi routine manual and service operations; routine manual and service operations; (2) technical and craft operations; (3) clerical and intermediate occupations; and (4) modern professional occupations; senior managers and administrators; middle and junior managers; traditional professional occupations. Those classed as in training or not in work or were coded as missing.

*Education*. Mothers’ and fathers’ highest educational qualification was recorded in 10 levels: (1) 1+ O-Levels, CSE’s or GCSEs at any grade, (2) 5+ O-Levels, CSE Grade One or GCSE School Certification, (3) 1+ A Level or AS Level, (4) 2+ A Level, or 4+ AS Levels or High School Certificate, (5) NVQ Level 1 – foundation GNVQ, (6) NVQ Level 2 – intermediate GNVQ, (7) NVQ Level 3 – advanced GNVQ, (8) NVQ Level 4-5, HNC or HND, (9) first degree (BA/BSc), and (10) higher degree (MA, PhD, PGCE). Other qualifications, overseas qualifications, and no qualifications were coded as missing.

### Table S17.1. Descriptives for BIB

|  | N | M | SD | Med | Min | Max | Skew | Kurtosis | S.E. |
| --- | --- | --- | --- | --- | --- | --- | --- | --- | --- |
| BIB Foundation stage profile (wave 2) | 13858 | 0.00 | 0.90 | 0 | -4.63 | 2.32 | -0.20 | 0.64 | 0.01 |
| BIB Father occupation (wave 1) | 8142 | 2.24 | 1.30 | 2 | 1 | 4 | 0.37 | -1.61 | 0.01 |
| BIB Father education (wave 1) | 5453 | 5.53 | 3.29 | 6 | 1 | 10 | 0.01 | -1.65 | 0.04 |
| BIB Mother education (wave 1) | 6755 | 5.30 | 3.02 | 6 | 1 | 10 | 0.08 | -1.43 | 0.04 |

### Table S17.2. Correlations for BIB

|  |  | 1 | 2 | 3 | 4 |
| --- | --- | --- | --- | --- | --- |
| 1 | BIB Foundation stage Profile (wave 2) | - |  |  |  |
| 2 | BIB Father occupation (wave 1) | .16 | - |  |  |
| 4 | BIB Father education (wave 1) | .08 | .47 | - |  |
| 5 | BIB Mother education (wave 1) | .15 | .29 | .50 | - |

## Growing Up in Scotland (Birth Cohort Two)

Growing up in Scotland was established in 2005 to monitor and evaluate children’s family services, with a focus on early years’ policy. Details of Birth Cohort 1 can be found on page 33. Birth Cohort 2, described below, tracked 6,127 children born in 2010 to 2011 (Bradshaw & Corbett, 2013).

## School Performance

**Sweep 3 (2015).** When the children were around 4.75 years old, two subscales of the British Ability Scales Version Three (BASIII; Elliott, Smith & McCulloch, 2011) were administered by an interviewer at the children’s home. Testing materials included the early years core booklet, early years record booklet, similarities response cards, and a CAPI tablet for scoring. While the assessments materials were almost identical to the BASII measures used in Birth Cohort 1, there were a small number of differences – for example in individual items, item order and stopping points. Only ability scores, not raw scores, were available for Birth Cohort 2.

*The Naming Vocabulary Subscale* assessed verbal skills and children’s expressive spoken language ability using 36 items. Children were shown a booklet with coloured pictures which they had to name. High scores reflected good expressive language skills, an ability to attach verbal labels to pictures, and retrieval of names from long-term memory. Children scored 1 point for a correct response and 0 points for an incorrect response. Instructions could be rephrased or repeated if the children asked or did not seem to understand. Blank cases were coded as missing.

*The Picture Similarity Subscale* assessed children’s reasoning ability using 33 items. Children were shown a row of four pictures or designs in a test booklet. They were required to find an appropriate fifth card which best matched the pictures or design. The relationships between pictures became increasingly complex. Children scored 1 point for a correct response and 0 points for an incorrect response. Blank cases were coded as missing.

### Family SES

**Sweep 1 (2011).** Interviewers visited the cohort members’ homes and conducted CAPI with children’s primary caregiver.

*Occupation*. Mothers’ and fathers’ occupational status was categorised using the NS-SEC: (1) managerial and professional occupations, (2) intermediate occupations, (3) small employers and own account workers, (4) lower supervisory and technical occupations, and (5) semi-routine and routine occupations. We recoded the scores, so that lower values indicated lower occupational status. Those who had never worked or did not give employment information were coded as missing.

*Education*. Mothers’ and fathers’ highest educational qualifications were recorded in four levels: (3) GCSE's, Standard Grades, NVQ Level 2 or below, (4) A -Levels, Highers, NVQ Level 3 or equivalent, (5) Higher National Certificate, Higher National Diploma, NVQ Level 4 or equivalent, (6) Degree, NVQ Level 5 or equivalent. We recoded the scores, so that lower values indicated a lower level qualification. Those with no information or other qualifications were coded as missing.

*Income*. Annual total household income before tax was banded in 9 levels: (1) Less than £5,200 pa, (2) £5,200 - 10,399 pa, (3) £10,400 - £15,599 pa, (4) £15,600 - £20,799 pa, (5) £20,800 - £25,999 pa, (6) £26,000 - £36,399 pa, (7) £36,400 - £51,999 pa, (8) £52,000 - £77,999 pa, and (9) £78,000 or more pa.

**Sweep 2 (2013).** CAPIs were conducted with the same respondent as in sweep 1, where possible; if not, another primary caregiver was interviewed.

*Occupation*. Mothers’ and fathers’ occupational status were assessed as described for sweep 1.

*Education*. Mothers’ and fathers’ highest education qualifications were assessed as described for sweep 1.

*Income*. Annual total household income before tax was assessed as described for sweep 1.

**Sweep 3 (2015).** CAPIs were conducted with the same respondent as in sweep 1, where possible; if not, another primary caregiver was interviewed.

*Occupation*. Mothers’ and fathers’ occupational status were assessed as described for sweep 1 & 2.

*Education*. Mothers’ and fathers’ highest education qualifications were assessed as described for sweep 1 & 2.

*Income*. Annual total household income before tax was banded in 17 levels: (1) Less than £3,999 pa, (2) £4,000 - £5,999 pa, (3) £6,000 - £7,999 pa, (4) £8,000 - £9,999 pa, (5) £10,000 - £11,999 pa, (6) £12,000 - £14,999 pa, (7) £15,000 - £17,999 pa, (8) £18,000 - £19,999 pa, (9) £20,000 - £22,999 pa, (10) £23,000 - £25,999 pa, (11) £26,000 - £28,999 pa, (12) £29,000 - £31,999 pa, (13) £32,000 - £37,999 pa, (14) £38,000 - £43,999 pa, (15) £44,000 - £49,999 pa, (16) £50,000 - £55,999 pa, and (17) £56,000 or more pa.

### Table S18.1. Descriptives for GUSBC2

|  | N | M | SD | Med | Min | Max | Skew | Kurtosis | S.E. |
| --- | --- | --- | --- | --- | --- | --- | --- | --- | --- |
| GUSBC2 BAS naming vocabulary (wave 5) | 4326 | 141.49 | 19.17 | 144 | 10 | 210 | -1.19 | 12.24 | 0.29 |
| GUSBC2 BAS picture similarity (wave 5) | 4314 | 108.13 | 13.52 | 1.07 | 10 | 146 | -0.53 | 6.43 | 0.21 |
| GUSBC2 Father occupation (wave 1) | 4861 | 3.28 | 1.67 | 3 | 1 | 4 | -0.21 | -1.64 | 0.02 |
| GUSBC2 Mother occupation (wave 1) | 5756 | 3.32 | 1.73 | 4 | 1 | 4 | -0.38 | -1.63 | 0.02 |
| GUSBC2 Father education (wave 1) | 4509 | 2.52 | 1.22 | 2 | 1 | 3 | 0.05 | -1.59 | 0.02 |
| GUSBC2 Mother education (wave 1) | 5709 | 2.61 | 1.24 | 3 | 1 | 3 | -0.08 | -1.62 | 0.02 |
| GUSBC2 Household income (wave 1) | 5409 | 5.53 | 2.18 | 6 | 1 | 8 | -0.26 | -0.98 | 0.03 |
| GUSBC2 Father occupation (wave 2) | 4114 | 3.37 | 1.65 | 4 | 1 | 4 | -0.30 | -1.58 | 0.03 |
| GUSBC2 Mother occupation (wave 2) | 4814 | 3.39 | 1.71 | 4 | 1 | 4 | -0.46 | -1.53 | 0.02 |
| GUSBC2 Father education (wave 2) | 3808 | 2.57 | 1.22 | 2 | 1 | 3 | -0.02 | -1.59 | 0.02 |
| GUSBC2 Mother education (wave 2) | 4712 | 2.67 | 1.23 | 3 | 1 | 3 | -0.16 | -1.59 | 0.02 |
| GUSBC2 Household income (wave 2) | 4370 | 5.97 | 2.06 | 6 | 1 | 8 | -0.41 | -0.75 | 0.03 |
| GUSBC2 Father occupation (wave 3) | 3698 | 3.40 | 1.64 | 4 | 1 | 4 | -0.33 | -1.56 | 0.03 |
| GUSBC2 Mother occupation (wave 3) | 4305 | 3.44 | 1.68 | 4 | 1 | 4 | -0.51 | -1.45 | 0.03 |
| GUSBC2 Father education (wave 3) | 3360 | 2.64 | 1.22 | 3 | 1 | 3 | -0.10 | -1.58 | 0.02 |
| GUSBC2 Mother education (wave 3) | 4098 | 2.82 | 1.20 | 3 | 1 | 3 | -0.35 | -1.47 | 0.02 |
| GUSBC2 Household income (wave 3) | 3992 | 12.51 | 4.35 | 14 | 1 | 16 | -0.71 | -0.65 | 0.07 |

### Table S18.2 Correlations for GUSBC2

|  |  | 1 | 2 | 3 | 4 | 5 | 6 | 7 | 8 | 9 | 10 | 11 | 12 | 13 | 14 | 15 | 16 |
| --- | --- | --- | --- | --- | --- | --- | --- | --- | --- | --- | --- | --- | --- | --- | --- | --- | --- |
| 1 | GUSBC2 BAS naming vocabulary (wave 5) | - |  |  |  |  |  |  |  |  |  |  |  |  |  |  |  |
| 2 | GUSBC2 BAS picture similarity (wave 5) | .34 | - |  |  |  |  |  |  |  |  |  |  |  |  |  |  |
| 3 | GUSBC2 Father occupation (wave 1) | .17 | .07 | - |  |  |  |  |  |  |  |  |  |  |  |  |  |
| 4 | GUSBC2 Mother occupation (wave 1) | .20 | .10 | .41 | - |  |  |  |  |  |  |  |  |  |  |  |  |
| 5 | GUSBC2 Father education (wave 1) | .10 | .05 | .54 | .34 | - |  |  |  |  |  |  |  |  |  |  |  |
| 6 | GUSBC2 Mother education (wave 1) | .17 | .10 | .40 | .55 | .45 | - |  |  |  |  |  |  |  |  |  |  |
| 7 | GUSBC2 Household income (wave 1) | .18 | .11 | .47 | .52 | .38 | .48 | - |  |  |  |  |  |  |  |  |  |
| 8 | GUSBC2 Father occupation (wave 2) | .18 | .08 | .91 | .39 | .52 | .40 | .46 | - |  |  |  |  |  |  |  |  |
| 9 | GUSBC2 Mother occupation (wave 2) | .20 | .10 | .40 | .90 | .33 | .56 | .51 | .38 | - |  |  |  |  |  |  |  |
| 10 | GUSBC2 Father education (wave 2) | .11 | .05 | .52 | .32 | .98 | .42 | .35 | .51 | .31 | - |  |  |  |  |  |  |
| 11 | GUSBC2 Mother education (wave 2) | .17 | .09 | .40 | .56 | .44 | .99 | .47 | .39 | .56 | .42 | - |  |  |  |  |  |
| 12 | GUSBC2 Household income (wave 2) | .18 | .11 | .45 | .50 | .38 | .48 | .73 | .46 | .52 | .37 | .48 | - |  |  |  |  |
| 13 | GUSBC2 Father occupation (wave 3) | .18 | .08 | .85 | .40 | .52 | .41 | .46 | .91 | .40 | .51 | .40 | .46 | - |  |  |  |
| 14 | GUSBC2 Mother occupation (wave 3) | .20 | .10 | .39 | .82 | .33 | .54 | .51 | 39 | .89 | .32 | 54 | .51 | .38 | - |  |  |
| 15 | GUSBC2 Father education (wave 3) | .12 | .05 | .51 | .33 | .97 | .42 | .37 | .51 | .33 | .98 | .42 | .37 | .53 | .33 | - |  |
| 16 | GUSBC2 Mother education (wave 3) | .17 | .10 | .39 | .53 | .42 | .96 | .44 | .39 | .54 | .40 | .95 | .44 | .40 | .53 | .42 | - |
| 17 | GUSBC2 Household income (wave 3) | .21 | .14 | .47 | .50 | .39 | .48 | .71 | .47 | .52 | .38 | .48 | .77 | .49 | .52 | .39 | .46 |

# References

Boyd, A., Golding, J., Macleod, J., Lawlor, D. A., Fraser, A., Henderson, J., ... & Davey Smith, G. (2013). Cohort profile: the ‘children of the 90s’—the index offspring of the Avon Longitudinal Study of Parents and Children. *International journal of epidemiology*, *42*(1), 111-127.

Bradshaw, P., & Corbett, J. (2013). *Growing up in Scotland: Birth Cohort 2, Sweep 1, User Guide.* Edinburgh: Scottish Centre for Social Research.

Bradshaw, P., Tipping, S., Marryat, L., & Corbett, J. (2007). *Growing Up in Scotland Sweep 1, 2005 User Guide*. Edinburgh: Scottish Centre for Social Research.

Brimer, M. A., & Dunn, L. M. (1962*). English Picture Vocabulary Tests: Test 1 (age range 5.0-8-11), Test 2 (age range 7.0-11.11), Pre-school Version (age range 3.0-4.11).* Educational Evaluation Enterprises.

Connelly, R., & Platt, L. (2014). Cohort Profile: UK Millennium Cohort Study (MCS). *International Journal of Epidemiology*, *43*(6), 1719-1725. doi: https://doi.org/10.1093/ije/dyu001

Elliott, C.D., Smith, P, and McCulloch, K. (1996). *British Ability Scales Second Edition (BAS II): Administration and Scoring Manual*. London: NFER-Nelson.

Elliott, C.D., Smith, P, and McCulloch, K. (1997). *British Ability Scales Second Edition (BAS II): Technical Manual*. London: NFER-Nelson.

Elliott, C.D., Smith, P, and McCulloch, K. (2011). *British Ability Scales Third Edition (BAS III): Administration and Scoring Manual.* London: NFER-Nelson.

Elliott, J., & Shepherd, P. (2006). Cohort profile: 1970 British Birth Cohort (BCS70). *International Journal of Epidemiology*, *35*(4), 836-843. doi: https://doi.org/10.1093/ije/dyl174

General Register Office. (1956). *Census 1951: Classification of occupations.*

Goodenough, F. L. (1926). Measurement of intelligence by drawings.

Goodenough, F.L., & Harris, D.B. (1963). *The Goodenough-Harris Drawing Test*. New York: Harcourt, Brace, and World.

Kalverboer, A.F. (1972). *A Profile Test for the Spatial-Constructive Development*. Lisse: Switz & Zeitlinger.

Koppitz, E. M. (1968).*Psychological evaluation of children's human figure drawings.* Grune & Stratton.

Leon, D. A., Lawlor, D. A., Clark, H., & Macintyre, S. (2006). Cohort Profile: the Aberdeen children of the 1950s study. *International Journal of Epidemiology*, *35*(3), 549-552. doi: https://doi.org/10.1093/ije/dyi319

Oliver, B. R., & Plomin, R. (2007). Twins' Early Development Study (TEDS): A multivariate, longitudinal genetic investigation of language, cognition and behavior problems from childhood through adolescence. *Twin Research and Human Genetics*, *10*(1), 96-105. doi: https://doi.org/10.1375/twin.10.1.96

Power, C., & Elliott, J. (2006). Cohort profile: 1958 British birth cohort (national child development study). *International Journal of Epidemiology*, *35*(1), 34-41. doi: https://doi.org/10.1093/ije/dyi183

Pringle, M. K., Butler, N., & Davie, R. (1966). *11,000 Seven Year Olds*. Longman, in association with National Children's Bureau.

Rimfeld, K., Malanchini, M., Spargo, T., Spickernell, G., Selzam, S., McMillan, A., ... & Plomin, R. (2019). Twins early development study: A genetically sensitive investigation into behavioral and cognitive development from infancy to emerging adulthood. Twin Research and Human Genetics, 1-6.

Rose, D., & Pevalin, D. J. (2001). *The national statistics socio-economic classification: Unifying official and sociological approaches to the conceptualisation and measurement of social class* (No. 2001-04). ISER Working Paper Series.

Rutter, M., Tizard, J. and Whitmore, K. (1970). *Education, Health and Behaviour.* London: Longman.

Schönbrodt, F. D., & Perugini, M. (2013). At what sample size do correlations stabilize?. *Journal of Research in Personality*, *47*(5), 609-612.

Schonell, F.J. (1971). *Reading and spelling tests: Handbook of instructions*. Edinburgh: Oliver and Boyd.

Scottish Executive. (2004) *A Curriculum for Excellence: The Curriculum Review Group*. Edinburgh, Scottish Executive.

Sharp, H., Pickles, A., Meaney, M., Marshall, K., Tibu, F., & Hill, J. (2012). Frequency of infant stroking reported by mothers moderates the effect of prenatal depression on infant behavioural and physiological outcomes. *PloS one*, *7*(10).

Standard Occupational Classification. (2000). Summary of Structure. *Office for National Statistics*

Taggart, B., Sylva, K., Melhuish, E., Sammons, P., & Siraj, I. (2015). *How pre-school influences children and young people's attainment and developmental outcomes over time.* Department of Education*.*

Taylor, A. M., Pattie, A., & Deary, I. J. (2018). Cohort profile update: the Lothian Birth Cohorts of 1921 and 1936. *International Journal of Epidemiology*, *47*(4), 1042-1042r. doi: https://doi.org/10.1093/ije/dyy022

Wadsworth, M., Kuh, D., Richards, M., & Hardy, R. (2006). Cohort profile: the 1946 national birth cohort (MRC National Survey of Health and Development). *International Journal of Epidemiology*, *35*(1), 49-54. doi: https://doi.org/10.1093/ije/dyi201

Watson, N., & Wooden, M. (2009). Identifying factors affecting longitudinal survey response. *Methodology of longitudinal surveys, 1,* 157-182.

Whalley, L. J., Murray, A. D., Staff, R. T., Starr, J. M., Deary, I. J., Fox, H. C., ... & Crawford, J. R. (2011). How the 1932 and 1947 mental surveys of Aberdeen schoolchildren provide a framework to explore the childhood origins of late onset disease and disability. *Maturitas*, *69*(4), 365-372. doi: 10.1016/j.maturitas.2011.05.010.

Wright, J., Small, N., Raynor, P., Tuffnell, D., Bhopal, R., Cameron, N., ... & Pickett, K. E. (2013). Cohort profile: The Born in Bradford multi-ethnic family cohort study. *International Journal of Epidemiology*, *42*(4), 978-991. doi: https://doi.org/10.1093/ije/dys112
